# Assessment of flomoxef combined with amikacin in a hollow-fibre infection model for the treatment of neonatal sepsis in low- and middle-income healthcare settings

**DOI:** 10.1093/jac/dkac323

**Published:** 2022-09-30

**Authors:** Christopher A Darlow, Laura McEntee, Adam Johnson, Nicola Farrington, Jennifer Unsworth, Ana Jimenez-Valverde, Bhavana Jagota, Ruwanthi Kolamunnage-Dona, Renata M A Da Costa, Sally Ellis, François Franceschi, Mike Sharland, Michael Neely, Laura Piddock, Shampa Das, William Hope

**Affiliations:** Antimicrobial Pharmacodynamics and Therapeutics, University of Liverpool, Liverpool Health Partners, Liverpool L69 7BE, UK; Antimicrobial Pharmacodynamics and Therapeutics, University of Liverpool, Liverpool Health Partners, Liverpool L69 7BE, UK; Antimicrobial Pharmacodynamics and Therapeutics, University of Liverpool, Liverpool Health Partners, Liverpool L69 7BE, UK; Antimicrobial Pharmacodynamics and Therapeutics, University of Liverpool, Liverpool Health Partners, Liverpool L69 7BE, UK; Antimicrobial Pharmacodynamics and Therapeutics, University of Liverpool, Liverpool Health Partners, Liverpool L69 7BE, UK; Antimicrobial Pharmacodynamics and Therapeutics, University of Liverpool, Liverpool Health Partners, Liverpool L69 7BE, UK; Antimicrobial Pharmacodynamics and Therapeutics, University of Liverpool, Liverpool Health Partners, Liverpool L69 7BE, UK; Department of Health Data Science, University of Liverpool, Liverpool Health Partners, Liverpool L69 3GF, UK; Global Antibiotic Research and Development Partnership, 15 Chemin Camille-Vidart, Geneva 1202, Switzerland; Global Antibiotic Research and Development Partnership, 15 Chemin Camille-Vidart, Geneva 1202, Switzerland; Global Antibiotic Research and Development Partnership, 15 Chemin Camille-Vidart, Geneva 1202, Switzerland; Paediatric Infectious Diseases Research Group, St George’s University of London, London SW17 0QT, UK; Department of Infectious Diseases, Children’s Hospital Los Angeles and the Keck School of Medicine, University of Southern California, Los Angeles, CA 90027, USA; Global Antibiotic Research and Development Partnership, 15 Chemin Camille-Vidart, Geneva 1202, Switzerland; Antimicrobial Pharmacodynamics and Therapeutics, University of Liverpool, Liverpool Health Partners, Liverpool L69 7BE, UK; Antimicrobial Pharmacodynamics and Therapeutics, University of Liverpool, Liverpool Health Partners, Liverpool L69 7BE, UK

## Abstract

**Background:**

Annual mortality from neonatal sepsis is an estimated 430 000–680 000 infants globally, most of which occur in low- and middle-income countries (LMICs). The WHO currently recommends a narrow-spectrum β-lactam (e.g. ampicillin) and gentamicin as first-line empirical therapy. However, available epidemiological data demonstrate high rates of resistance to both agents. Alternative empirical regimens are needed. Flomoxef and amikacin are two off-patent antibiotics with potential for use in this setting.

**Objectives:**

To assess the pharmacodynamics of flomoxef and amikacin in combination.

**Methods:**

The pharmacodynamic interaction of flomoxef and amikacin was assessed in chequerboard assays and a 16-arm dose-ranged hollow-fibre infection model (HFIM) experiment. The combination was further assessed in HFIM experiments mimicking neonatal plasma exposures of clinically relevant doses of both drugs against five Enterobacterales isolates with a range of flomoxef/amikacin MICs.

**Results:**

Flomoxef and amikacin in combination were synergistic in bacterial killing in both assays and prevention of emergence of amikacin resistance in the HFIM. In the HFIM assessing neonatal-like drug exposures, the combination killed 3/5 strains to sterility, (including 2/5 that monotherapy with either drug failed to kill) and failed to kill the 2/5 strains with flomoxef MICs of 32 mg/L.

**Conclusions:**

We conclude that the combination of flomoxef and amikacin is synergistic and is a potentially clinically effective regimen for the empirical treatment of neonatal sepsis in LMIC settings and is therefore suitable for further assessment in a clinical trial.

## Introduction

Despite improvement in neonatal mortality in recent decades,^1^ neonatal sepsis continues to contribute significant global mortality, with an estimated 430 000–680 000 deaths per annum, with a majority of deaths occurring in low- and middle-income countries (LMICs).^2–4^ The WHO currently recommends a narrow-spectrum β-lactam (e.g. benzylpenicillin or ampicillin) in combination with gentamicin for the first-line empirical treatment of neonatal sepsis, with a third-generation cephalosporin (e.g. cefotaxime, ceftriaxone) recommended as second line.^5,6^

A recent prospective observational neonatal sepsis study in South Asia and sub-Saharan Africa demonstrated resistance rates in Gram-negative bacteria of 95%, 83% and 60% to ampicillin, cefotaxime and gentamicin, respectively.^7^ Another prospective study in New Delhi demonstrated 56% of Gram-negative bacteria being resistant to ≥3 classes of broad-spectrum antibiotics (i.e. extended-spectrum cephalosporins, piperacillin/tazobactam, fluoroquinolones, aminoglycosides and carbapenems) and 38% methicillin resistance rates in *Staphylococcus aureus.*^8^ A similar pattern of high resistance rates is seen in other retrospective studies.^9–15^ Alternative antimicrobial regimens for empirical treatment of neonatal sepsis in LMIC settings are urgently required.

Alternative antimicrobial agents for empirical treatment of neonatal sepsis in LMICs should meet the following criteria: (1) be effective against relevant pathogens with resistance mechanisms complicating the current WHO-recommended regimen; (2) be safe and well tolerated; (3) licensed for use in neonates (or with experience of extensive usage); and (4) be affordable.^16^

Amikacin is an aminoglycoside with molecular modifications that render it stable to aminoglycoside-modifying enzymes that inactivate gentamicin.^17–19^ Flomoxef is an oxacephem β-lactam with stability to degradation by non-AmpC ESBLs.^19,20^ Both agents fulfil the above criteria, and each have been demonstrated to have *in vitro* synergy in combination with fosfomycin.^21,22^ We therefore studied the potential utility of these agents in combination by assessing their *in vitro* activity, the presence and magnitude of any pharmacodynamic (PD) interaction using dynamic *in vitro* models, and assessed the potential utility of this candidate combination regimen using clinically relevant drug exposures.

## Materials and methods

The methodology is similar to that of previous experimental work assessing the pharmacodynamics of antibiotics in neonates in a pre-clinical setting.^21^

### Antimicrobial agents

Pure compounds of flomoxef (Shionogi, Osaka, Japan) and amikacin (Sigma–Aldrich, St Louis, USA) were obtained for all *in vitro* experiments. Both agents were stored at 2°C–8°C in anhydrous form and were prepared in sterile distilled water prior to any experiment.

### Media

CAMHB (Sigma–Aldrich, St Louis, USA) was used as the primary medium in all experiments. Mueller–Hinton agar (MHA) was used in all agar plates. Commercially pre-prepared 20 mL round MHA plates (Fisher Scientific, Waltham, USA) or self-prepared 50 mL square MHA plates (MHA from Sigma–Aldrich; square plates from VWR, Radnor, USA) were used in all experiments. For drug-containing plates, MHA was supplemented with antibiotic and prepared within each antibiotic’s stability limits and stored at 2°C–8°C (1 week for both agents). Drug concentrations in agar were four times the MIC of the specific bacterial strain used in a given experiment.

### Bacterial isolates

Isolates were supplied by JMI Laboratories, IHMA, PHE, LGC standards, University of Birmingham, University of Oxford and Royal Liverpool University Hospital. For the initial non-dynamic *in vitro* experiments, a collection of strains was collated representing a range of possible neonatal sepsis bacterial pathogens and resistance mechanisms in an antimicrobial resistance (AMR)-prevalent environment. In total, this included 10 strains of each of the following: *Streptococcus agalactiae*, MRSA, *Escherichia coli* and *Klebsiella pneumoniae*. All Enterobacterales were ESBL (nine *E. coli* and nine *K. pneumoniae* strains) or carbapenemase producers (one *E. coli* and one *K. pneumoniae* strain). Some of these strains were used also in the hollow-fibre infection model (HFIM) based on their MICs. Full details of the isolates are detailed in Table S1, available as Supplementary data at *JAC* Online, with isolates used in the HFIM detailed in Table 1. All isolates were stored in glycerol at −80°C and subcultured onto two MHA plates for 18–24 h at 37°C prior to each experiment. In each experiment, colonies were suspended in PBS to MacFarland standard 0.5 (1 × 10^8^ cfu/mL) and diluted to the target concentration.

**Table 1.** Details of strains used in the HFIM experiments

Strain ID	Species	Resistance mechanisms	Flomoxef MIC (mg/L)	Amikacin MIC (mg/L)
ST195	*E. coli*	CTX-M-14, *aph(3′)*, *aac(3)*, TEM-OSBL, *mdf*(A)	1	4
SPT 731	*E. coli*	CTX-M-1, TEM, ST131, O25b	0.125	16
I1025	*E. coli*	*mdf*(A), *ampC* promoter mutation	8	4
1203217	*K. pneumoniae*	SHV-12, CTX-M-9, OXA-48	0.5	1
1280740	*K. pneumoniae*	SHV-OSBL, TEM-OSBL, CTX-M-15, DHA-1	32	4
1256506	*K. pneumoniae*	SHV-OSBL; TEM-OSBL; CTX-M-2; CMY-2	32	2

### Antimicrobial susceptibility testing

Amikacin and flomoxef MICs for a panel of representative neonatal sepsis bacterial pathogens were determined using the EUCAST broth microdilution methodology.^23^*E. coli* ATCC 25922 or *S. aureus* ATCC 29213 were used as quality control isolates in all experiments, interpreted using QC values from EUCAST and the Japanese Society of Chemotherapy.^24,25^ The antibiotic gradient strip assay method was used for amikacin MIC determination from isolates from the hollow-fibre experiment. Briefly, an inoculum of the isolate was made using a suspension of a sweep of colonies into PBS to a McFarland standard of 0.5. A lawn of the inoculum was plated onto an MHA plate and an antibiotic gradient strip (Etest, bioMérieux, Marcy-l’Étoile, France) placed on the plate, which was subsequently incubated for 18–24 h at 37°C before reading.

### In vitro PD assays

Chequerboard assays were used on selected strains to assess the PD interaction of the flomoxef/amikacin combination, using a similar method as described previously.^21^ Strains were selected based on having MICs ≤32 mg/L and >0.0625 mg/L to both amikacin and flomoxef. A total of 100 μL of antimicrobials in sterile distilled water was added to an 8 × 8 grid on a 96-well plate, with concentration gradients created with 1:2 serial dilutions along each axis, with the final row/column having 0 mg/L of the appropriate drug. Each plate was assembled bespoke to each strain, with the maximum concentration of antimicrobial being 4 ×  the MIC for that strain. One hundred microlitres of a 1 × 10^6^ cfu/mL inoculum was added to each well of the prepared chequerboard. The well containing 0 mg/mL of each drug acted as the positive control; an additional row of blank MHB on the plate acted as negative control. Plates were incubated for 18–24 h at 37°C before being read by an optical densitometer (Varioskan, Thermo Fisher) at 600 nm. Plates were considered valid if the MIC on the monotherapy rows of the chequerboard were within one dilution of previously determined MICs, the negative controls had no growth, and quantification of the inoculum was within 6 × 10^5^–14 × 10^5^ cfu/mL.

Raw OD readings were normalized to that of the positive control. The readouts were then modelled using Greco’s model of drug synergy using ADAPT 5.^26,27^ The model produced an interaction parameter, α, with 95% CI characterizing the PD interaction; which was interpreted as follows: a lower bound of the CI > 0 indicates synergy; an upper bound of the CI < 0 indicates antagonism; a CI containing 0 indicates additivity only. Meta-analysis was performed on the output of the model between individual strains using the R package ‘Metafor’.^28^

### Hollow-fibre infection model

The HFIM is a well-established dynamic model simulating the PD effect of antimicrobials with physiological dynamic concentrations.^29^ The HFIM method was used largely as described previously.^30^ Briefly, each arm in the HFIM is set up as demonstrated in Figure S1; monotherapy arms omitted the supplementary compartments. CAMHB was pumped into the central compartment at a rate set to simulate a physiological clearance rate for the drug, with all media in the central compartment above 300 mL removed via an elimination pump. The target-simulated half-lives for amikacin and flomoxef were 7 and 2.3 h respectively. The neonatal half-lives of both drugs were sourced from the respective SPCs^31,32^ and confirmed with other published neonatal clinical pharmacokinetic (PK) data.^19^ To account for the difference in clearance between flomoxef and amikacin, supplementary compartments were set up according the principles laid out by Blaser.^33^ Protein binding is negligible for both drugs,^34,35^ so no adjustment in the administered dosage to account for this was necessary. Biological and technical replicates were not performed due to cost, as is the standard in other published HFIM experiments.^36^

Preliminary monotherapy experiments were performed with the ESBL-producing ST195 *E. coli* strain (flomoxef MIC 0.125 mg/L; amikacin MIC 4 mg/L; supplied by the University of Birmingham).^37^ PK and PD outputs of these experiments were modelled using Pmetrics^38^ and parameters simulated using ADAPT^27^ to determine the flomoxef and amikacin doses required to achieve EC_20_, EC_50_ and EC_80_ in terms of bactericidal effect within the HFIM. A 16-arm HFIM experiment was performed using a 4 × 4 dosing matrix using these three doses and no dose for both antibiotics in combination. The experiment was run over 96 h, with a target initial inoculum of 1 × 10^6^ cfu/mL inoculated into the hollow-fibre cartridges. Doses of flomoxef were administered every 12 h to the primary central compartment only; amikacin doses were administered to the primary and supplementary central compartments every 24 h.

PK samples were taken for bioanalysis at four timepoints [pre-dose and 1, 4 and 12 h (for flomoxef)/24 h (for amikacin) post-dose] in dosing windows on Days 1 and 3 of the experiment, with drug concentrations determined via an LC-MS/MS bioanalysis methodology (see Text S1 in Supplementary data for full details). Inoculum samples were taken from each hollow-fibre cartridge at four timepoints (pre-first dose and 2, 4 and 6 h post-first dose) during the first 24 h, then once daily before administration of any doses until the 96 h timepoint. Inoculum concentrations were determined using the track dilution method,^39^ plated onto three MHA plates: one drug-free and two containing either flomoxef or amikacin. An additional 100 μL of the original inoculum was plated onto a drug-free MHA plate to lower the limit of detection for total bacterial quantification (i.e. to 10 cfu/mL). Plates were then incubated at 37°C for 18–24 h for drug-free plates, and 42–48 h for drug-containing plates. After incubation, colonies were counted for at least two dilutions and the cfu/mL of the original inoculum calculated. MICs from any viable colonies from each arm on the final timepoint were determined via antibiotic gradient strip assay for amikacin, and broth microdilution method for flomoxef.

Further HFIM experiments were performed, assessing the effect of clinically relevant flomoxef and amikacin doses leading to neonatal-like time–concentration profile alone and in combination against five Enterobacterales isolates with different flomoxef and amikacin MICs (Table 1). PK profiles of flomoxef and amikacin were designed to have half-lives of 2.3 and 7 h, with *C*_max_ values of 50 and 40 mg/L, respectively, to reflect the median neonatal time–concentration profile following an administration of 20 mg/kg IV dose of flomoxef^31^ and 15 mg/kg amikacin.^40^ These were determined from the sources used to determine the half-life, as described earlier. Each individual experiment consisted of four arms; monotherapy arms for both flomoxef and amikacin, a combination therapy arm and an untreated control. Each experiment lasted 7 days to reflect the typical treatment course of neonatal sepsis. Four PK samples [pre-dose and 1, 4 and 12 h (for flomoxef)/24 h (for amikacin) post-dose], were taken in each of three dose intervals distributed evenly throughout the experiment (after the first doses administered on experiment Days 1, 3 and 5 or Days 1, 4 and 6 depending on logistical constraints). Four inoculum samples were taken in the first 24 h (pre-first dose and 2, 4 and 6 h post-first dose) and once every 24 h thereafter. These samples were quantified on drug-free, flomoxef-containing and amikacin-containing square MHA plates. MICs from any viable colonies from each arm on the final timepoint were determined via antibiotic gradient strip assay (for amikacin) or broth microdilution MIC methodology (for flomoxef).

Suspected sterility of individual HFIM arms was confirmed at the end of the experiment by centrifugation of contents of the hollow-fibre cartridge at 3000 rpm for 10 min. The supernatant was discarded, and remainder of the sample resuspended in 1 mL MHB before being plated onto MHA plates and incubated for 24 h at 37°C.

### Modelling

Population PK models were constructed using the PK and PD outputs of the 16-arm HFIM experiment using the population PK program Pmetrics using a non-parametric adaptive grid (NPAG) estimation routine^38^ and a structural model was based on Greco’s models of pharmacological synergy^26^ (see Text S2 for details).

## Results

### In vitro susceptibility testing

The flomoxef and amikacin MICs were determined for a panel of strains representative of pathogens with identified resistance mechanisms pertinent to LMICs (Table 2). The modal flomoxef MIC was 0.25 mg/L. Of the six Enterobacterales strains with flomoxef MICs ≥8 mg/L, 3/6 carried a plasmid-borne gene encoding an AmpC enzyme (e.g. CMY-II); 1/6 was a carbapenemase (KPC3) producer; 1/6 carried an AmpC promoter mutation; and 1/6 had no identified relevant resistance mechanism. The flomoxef MICs were ≤0.5 mg/L for all *Streptococcus agalactiae*, and ≤4 mg/L for 9/10 MRSA strains. The modal MIC for amikacin was 4 mg/L (excluding the intrinsically amikacin-resistant *S. agalactiae*, which had a modal amikacin MIC of >32 mg/L).

**Table 2.** Flomoxef (top) and amikacin (bottom) MIC distributions for panel of 40 representative bacterial strains

Bacterial species	Flomoxef MIC (mg/L)										
	≤0.0625	0.125	0.25	0.5	1	2	4	8	16	32	>32
*E. coli*	1	4	2	1	—	—	—	1	—	—	1
*K. pneumoniae*	—	1	4	1	—	—	—	1	—	2	1
MRSA	—	—	1	1	1	1	5	—	1	—	—
*S. agalactiae*	1	1	7	1	—	—	—	—	—	—	—

Bacterial species	Amikacin MIC (mg/L)										
	≤0.0625	0.125	0.25	0.5	1	2	4	8	16	32	>32
*E. coli*	—	—	—	—	—	1	3	2	3	1	—
*K. pneumoniae*	—	—	—	—	1	3	2	2	—	—	2
MRSA	—	—	—	—	—	4	3	—	—	—	3
*S. agalactiae*	—	—	—	—	—	—	—	—	1	1	8

### In vitro drug–drug interaction modelling

**Figure 1.**
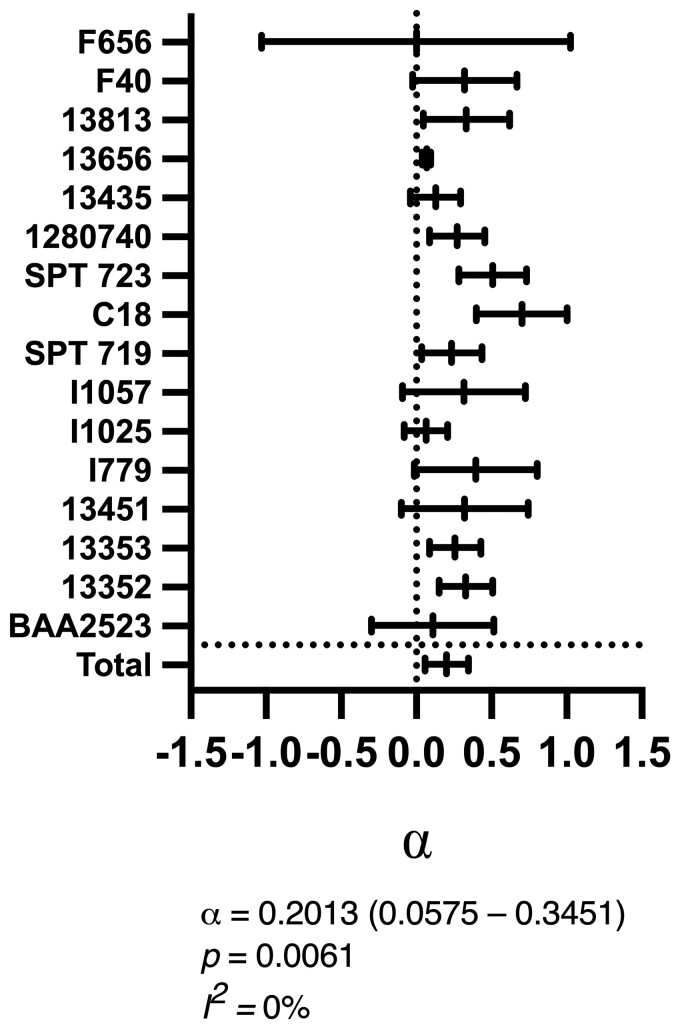
Fitted α values from the Greco model using chequerboard assays outputs for 16 strains. A total summary statistic using a meta-analysis of the 16 strains is demonstrated in the final row ('Total') with numerical values given above the figure.

Static chequerboard assays were performed on strains with both amikacin and flomoxef MICs >0.0625 mg/L and ≤32 mg/L (*n* = 16). A mathematical model of drug interaction originally described by Greco^26^ was fitted to the observed data to estimate the nature and magnitude of the PD interaction for each strain (Figure 1).

The combination demonstrated evidence of synergy for 8/16 strains (i.e. α and the 95% CI were >0). For the remaining 8/16 strains, the combination was additive (i.e. the 95% CI of α included 0). A meta-analysis of the individual strains demonstrated low heterogeneity between strains and species (*I^2^ *= 0%) with a summary value of α = 0.2013 (95% CI 0.0575–0.3451), indicating an overall synergistic interaction.

### PD interaction of flomoxef and amikacin

In preliminary HFIM experiments, the drug exposures of both agents that produced the EC_20_, EC_50_, and EC_80_ (quantified in terms of maximal bacterial kill) against the CTX-M-14-producing *E. coli* ST195 (flomoxef MIC 0.125 mg/L, amikacin MIC 4 mg/L) were determined as *f*AUC_0–24_ of 15, 65 and 120 mg·h/L for flomoxef (with *C*_max_ values of 2, 8.5 and 15 mg/L) and 45, 190 and 375 mg·h/L for amikacin (with *C*_max_ values of 5, 20 and 40 mg/L).

**Figure 2.**
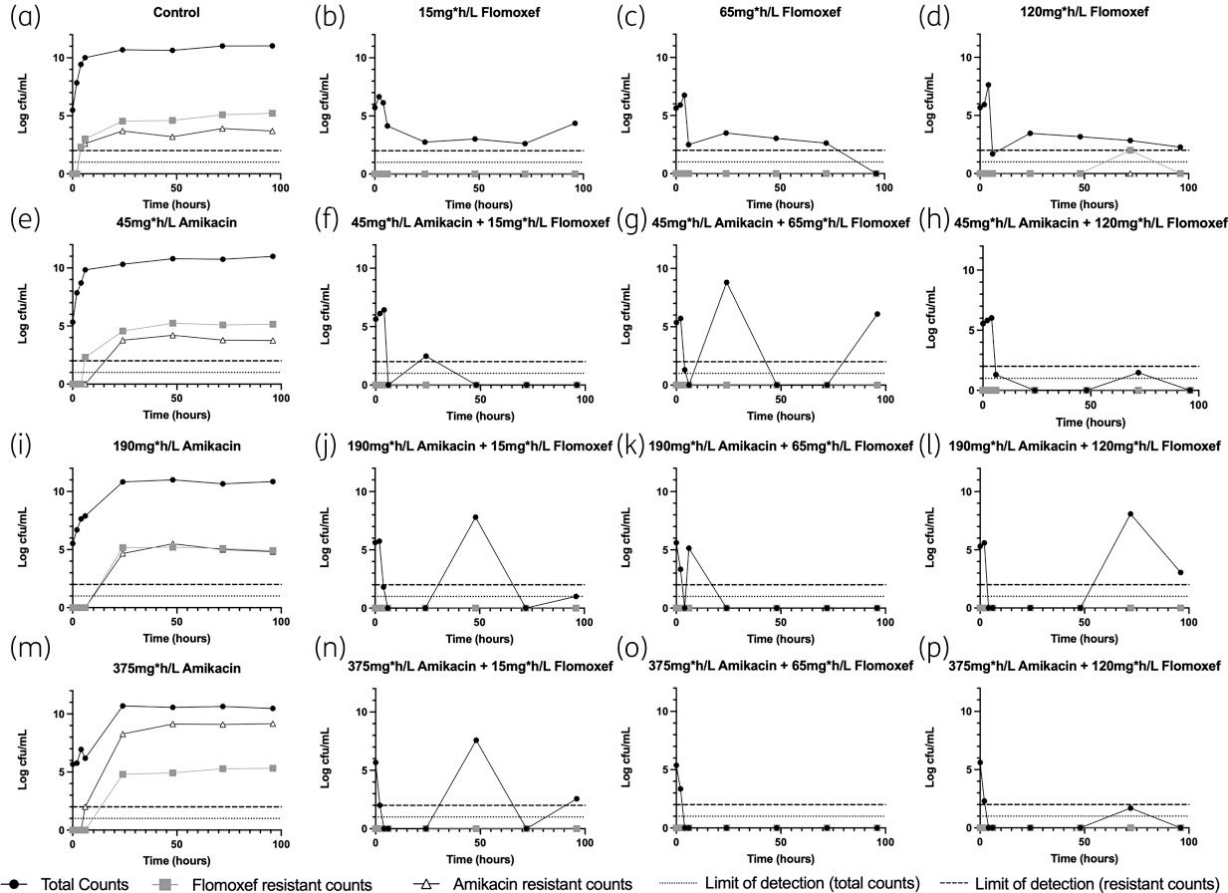
PD output of 16-arm flomoxef/amikacin combination experiment. Arms a-p represent individual hollow fibre experimental arms recieving the labelled exposure of each drug, given as *f*AUC_0-24_.

These EC_20_, EC_50_, and EC_80_ exposures for both drugs were used in a 16-arm 4 × 4 matrix, representing all possible monotherapy and combination regimens to explore the PD interaction between the two agents (Figure 2). Increasing exposures of flomoxef (as monotherapy) resulted in rapid bacterial killing in the first 24 h. However, none of the arms became sterile for the duration of the experiment [Figure 2(b–d)], although emergence of flomoxef resistance was not observed in the arm despite this. Increasing amikacin monotherapy saw an exposure-dependent decline of bacterial growth and progressive exposure-dependent emergence of amikacin resistance [Figure 2(e, i and m)]. All combination arms [Figure 2(f–h; j–l; n–p)] resulted in a greater magnitude of bacterial kill compared with comparable monotherapy doses without emergence of resistance to either amikacin or flomoxef.

A PD interaction model based on the model described by Greco^26^ was fitted to the data. The mean and median parameter estimates, and their dispersions, are summarized in Table 3. The coefficient of determination values for observed-versus-individual predicted values (using mean parameter estimates) were 0.985 (free flomoxef concentrations), 0.981 (free amikacin concentrations), 0.858 (total bacterial count), 0.875 (flomoxef-resistant bacterial count) and 0.923 (amikacin-resistant bacterial count). The mean α interaction parameter values were 54.96 (95% credibility interval 47.69–74.50) for bacterial kill and 35.66 (95% credibility interval 11.05–53.78) for prevention of amikacin resistance emergence. Modelled experimental time–concentration and PD profiles can be seen in Figures S2 and S3. An interpretable value of α could not be estimated for prevention of flomoxef resistance given the lack of flomoxef resistance emergence in flomoxef-containing arms.

**Table 3.** Parameter value estimates with 95% credibility interval from HFIM PK/PD interaction model

Parameter	Mean	Median	95% credibility interval
*V*1 (L)	0.308	0.285	0.283–0.360
*V*2 (L)	0.316	0.311	0.274–0.357
CL1 (L/h)	0.077	0.074	0.071–0.088
CL2 (L/h)	0.025	0.025	0.025–0.027
Kgs	1.66	1.53	1.50–1.76
Kks	4.38	4.61	4.35–4.95
E50_1_s (mg/L)	5.82	5.21	4.62–9.45
E50_2_s (mg/L)	14.19	12.32	10.82–15.66
α_s_	54.96	54.38	47.69–74.50
Kgr1	0.83	0.68	0.46–1.35
Kkr1	3.24	3.02	1.73–4.24
E50_1_r1 (mg/L)	32.43	32.15	26.80–34.32
α_r1_^a^	17.91	15.97	13.40–30.00
Kgr2	0.85	0.89	0.40–0.91
Kkr2	3.54	2.75	2.24–5.00
E50_2_r2 (mg/L)	30.39	27.84	25.00–36.59
α_r2_	35.66	44.07	11.05–53.78
H1s	0.81	0.54	0.49–0.80
H2s	2.63	2.44	1.73–2.45
H1r1	0.37	0.13	0.13–0.22
H2r2	1.59	1.32	1.12–1.84

Kg, bacterial growth constant; Kk, bacterial kill constant; E50, Concentration of drug achieving 50% of efficacy; α, interaction parameter; H, Hill constant. Parameter suffices are defined as follows; 1, relating to flomoxef; 2, relating to amikacin; s, relating to WT bacterial population; r1, relating to ‘flomoxef-resistant’ bacterial population; r2, relating to ‘amikacin-resistant’ bacterial population.

a α_r1_ is shown here for completeness, but given the lack of flomoxef resistance emerging in flomoxef containing arms, this value cannot be reliably interpreted.

### Assessment of flomoxef and amikacin using neonatal regimens

The pharmacodynamics of the flomoxef/amikacin combination replicating exposures of candidate neonatal regimens (i.e. IV 15 mg/kg q24h for amikacin and IV 20 mg/kg q12 h for flomoxef)^31,32^ were studied using five Enterobacterales strains with a range of flomoxef and amikacin MICs (Table 1).

**Figure 3.**
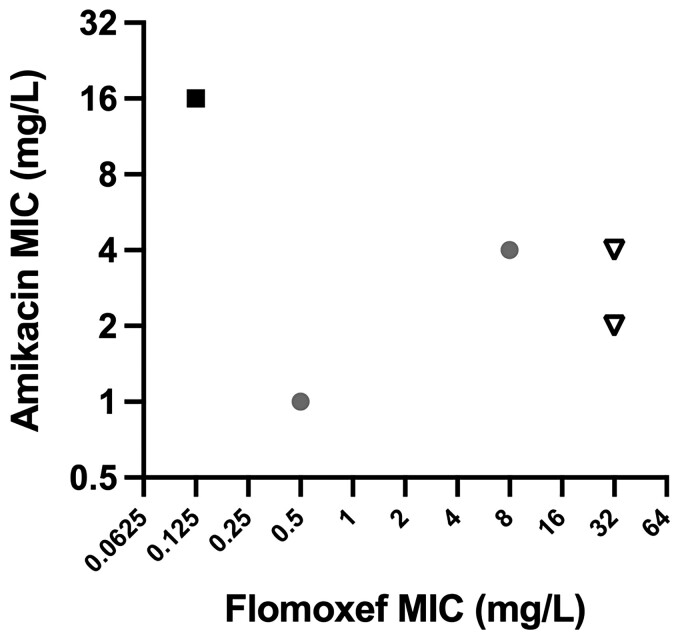
Summary of the outcome of the HFIM experiments replicating neonatal regimens of flomoxef and amikacin alone and in combination. ‘Success’ is defined as the achievement of sterility. Filled square = ‘success’ achieved by flomoxef monotherapy and combination regimens; filled circles = ‘success’ with combination regimen alone; open inverted triangle = failure to achieve sterility in all arms.

The final end-experiment PD outcomes from each arm are shown in Figure 3 (modelled time–concentration profiles are shown in Figure S4 with full PD outputs shown in Figures S5–S9). Flomoxef monotherapy resulted in sterilization of *E. coli* strain SPT 731 (flomoxef MIC 0.125 mg/L) but not for all other strains. Amikacin monotherapy failed to sterilize any strain. The combination regimen sterilized 2/4 strains not killed by either monotherapy. Neither of the two strains with a flomoxef MIC value of 32 mg/L were sterilized by the combination.

Colonies retrieved from end-experiment non-sterile arms following use of a flomoxef- or amikacin-containing regimen (i.e. either monotherapy or combination) had an increase in MIC by ≥4-fold to the respective agent.

## Discussion

In our experimental work, we have demonstrated that flomoxef and amikacin in combination provide a synergistic bactericidal effect (Figures 2 and S5–S9) and extends the spectrum of activity to strains that would otherwise not be successfully killed by monotherapy with either agent (Figures S6 and S7). The combination also synergistically prevents emergence of amikacin resistance, although we did not determine the effect of amikacin on protecting flomoxef resistance.

The pharmacodynamics of amikacin monotherapy in the HFIM is similar to previous work.^21^ Its relatively poor performance when administered as monotherapy may result from the emergence of small-colony variants at a greater rate *in vitro* than *in vivo*.^41^ These experimental data also support the recent downward revision of amikacin breakpoints by EUCAST and the new recommendation to avoid use of amikacin (and other aminoglycosides) as monotherapy in systemic infections.^42^

As monotherapy, flomoxef was not able to cause any overall bacterial kill in strains with significant production of AmpC. Significant kill and sterilization occurred only in strains with flomoxef MICs ≤0.5 mg/L. Combined with results from similar previous HFIM experiments,^22^ flomoxef monotherapy sterilized 4/5 strains with flomoxef MICs ≤0.5 mg/L, all without significant AmpC production. This experience is consistent with adult clinical data of flomoxef against non-AmpC ESBL-producing bacteria.^43^ The usefulness of the combination regimen over flomoxef monotherapy will therefore be primarily determined by local resistance epidemiology.

Flomoxef resistance is primarily driven by the production of AmpC enzymes and carbapenemases in Enterobacterales.^44^ Resistance to flomoxef also results from one or more additional mechanisms including porin loss and, most likely, efflux, as with other β-lactams. However, the specific characterization of these mechanisms for flomoxef is limited and their contribution to resistance requires further study. Where characterizations are made (e.g. loss of OmpF or OmpK35 porins), the effect on flomoxef MIC is relatively minor.^45,46^ Nevertheless, it remains likely the overall efficacy of flomoxef monotherapy will be directly related to the epidemiology of AmpC and carbapenemases.

Many Gram-negative bacteria contain chromosomal *ampC* genes.^47^ In the so-called ESCPM organisms (*Enterobacter* spp., *Serratia* spp., *Citrobacter freundii, Providencia* spp. and *Morganella* spp.) these chromosomal genes are quickly derepressed to produce clinically significant levels of AmpC. In other Enterobacterales, chromosomal *ampC* is either absent (e.g. *Klebsiella* spp.), or poorly expressed (e.g. *E. coli*).^47^ The latter group can acquire mutations in *ampC* promotors leading to clinically significant AmpC production. Additionally, all Enterobacterales can acquire plasmid-borne *ampC* (e.g. *bla*_CMY_ or *bla*_DHA_), which causes clinically significant production of AmpC.^47^

The recent BARNARDS study showed ESCPM organisms accounting for 247/1038 (23.8%) of Gram-negative bacteria and 247/2483 (9.9%) of total isolated bacteria from LMIC neonatal sepsis patients (these rates are likely overestimates of the true prevalence due to outbreaks of clonal *Serratia* spp. at one of the study sites).^7,48^ Plasmid-mediated *ampC* genes and carbapenemase genes were present in a further 17/258 (6.6%) and 69/258 (26.7%) of total *K. pneumoniae* isolates and 2/75 (2.7%) and 3/75 (4.0%) of total *E. coli* isolates, respectively. WGS data from the NeoOBS study suggest plasmid-borne *ampC* carriage rates of 7% and 2.5% in isolated *K. pneumoniae* and *E. coli* strains, respectively.^49^ Whilst there is regional variation, these point estimates suggest that the prevalence of AmpC-producing strains is high enough in these LMIC settings that a combination of flomoxef with amikacin would be beneficial compared with flomoxef monotherapy for the empirical treatment of neonatal sepsis in these settings.

There are several considerations for using this combination regimen. As monotherapy, both flomoxef and amikacin are safe and well tolerated.^19^ Whilst it is likely that combination therapy will be safe (given the experience of both agents as monotherapy, and the experience of ampicillin and gentamicin in combination), it is possible there may be unanticipated drug–drug interactions and potentiated toxicities when the drugs are used in combination. Additionally, the CSF penetration is relatively low for both agents, with neonatal CSF partition coefficients of 0.05 and 0.1 estimated for flomoxef and amikacin, respectively.^50,51^ These numbers compare favourably with the WHO regimen of ampicillin and gentamicin (CSF partition coefficients of ~0 for gentamicin and 0.05–0.1 for ampicillin),^52–54^ but our work did not model the pharmacokinetics within CSF, nor the pharmacodynamics within the CNS, and we therefore cannot comment on the efficacy of this regimen for the treatment of CNS infections.

There are potential limitations of this work. First, the HFIM does not contain or replicate immunological effectors. Secondly, the inoculum that was used in the HFIM is higher than that found in neonatal sepsis (10^5^–10^6^ cfu/mL compared with the estimated bacterial density in neonatal sepsis of 10^0^–10^3^ cfu/mL).^55,56^ Both limitations are likely to underestimate the predicted *in vivo* effect of the combination regimen. A smaller bacterial inoculum and present immune effectors (even if immature) in individuals with neonatal sepsis would likely lead to a greater overall effect of the regimen than seen *in vitro* here. The conclusions drawn from these experiments are therefore conservative and the HFIM likely represents a worst-case scenario.

Overall, we conclude that this experimental study has demonstrated this combination regimen is synergistic, in terms of both bactericidal effect and protection against amikacin resistance, and potentially efficacious in the empirical treatment of neonatal sepsis caused by ESBL-producing Enterobacterales species. Consequently, we believe this regimen should be considered for clinical assessment as empirical treatment of MDR neonatal sepsis in LMIC settings, where the current standard of care has become decidedly suboptimal.^21,30^

## Supplementary Material

dkac323_Supplementary_Data

## Data Availability

The programs ADAPT and Pmetrics are publicly available, with instructions, at https://bmsr.usc.edu/software/adapt/ and http://www.lapk.org/pmetrics.php, respectively.

## Transparency declarations

Shionogi provided the flomoxef material free of charge, but provided no additional funding. W.H. holds or has recently held research grants with UKRI, National Institutes of Health, National Institute of Health Research, F2G, Spero Therapeutics, Antabio, Pfizer, Bugworks, Phico Therapeutics, BioVersys, GARDP and NAEJA-RGM. He is (or has recently been) a consultant for Appili Therapeutics, F2G, Spero Therapeutics, NAEJA-RGM, Centauri, Pfizer, Phico Therapeutics and VenatoRx. He is a member of the Scientific Advisory Committee for GARDP and the Specialty National Co-lead for Infectious Diseases for the National Institute of Health Research (NIHR). S.D. holds or has recently held research grants with F2G, Spero Therapeutics, Antabio, Pfizer, Bugworks, Phico Therapeutics, BioVersys, GARDP and NAEJA-RGM. She is (or has recently been) a consultant for Spero Therapeutics, Centauri, Pfizer and CARBX. She is a member of EUCAST. There is nothing further to declare for all other authors.

## Supplementary data

Table S1, Figures S1 to S9 and Texts S1 to S2 are available as Supplementary data at *JAC* Online.

## References

[1] Lawn JE , Blencowe H, Oza S et al Every newborn: progress, priorities, and potential beyond survival. Lancet 2014; 384: 189–205. 10.1016/S0140-6736(14)60496-7 24853593

[2] Oza S , Lawn JE, Hogan DR et al Neonatal cause-of-death estimates for the early and late neonatal periods for 194 countries: 2000–2013. Bull World Health Organ 2015; 93: 19–28. 10.2471/BLT.14.139790 25558104 PMC4271684

[3] Fleischmann-Struzek C , Goldfarb DM, Schlattmann P et al The global burden of paediatric and neonatal sepsis: a systematic review. Lancet Respir Med 2018; 6: 223–30. 10.1016/S2213-2600(18)30063-8 29508706

[4] Seale AC , Blencowe H, Manu AA et al Estimates of possible severe bacterial infection in neonates in sub-saharan Africa, south Asia, and Latin America for 2012: a systematic review and meta-analysis. Lancet Infect Dis 2014; 14: 731–41. 10.1016/S1473-3099(14)70804-7 24974250 PMC4123782

[5] Fuchs A , Bielicki J, Mathur S et al Reviewing the WHO guidelines for antibiotic use for sepsis in neonates and children. Paediatr Int Child Health 2018; 38 Suppl 1: S3–15. 10.1080/20469047.2017.1408738 29790842 PMC6176768

[6] WHO . Pocket book of hospital care for children. Second edition. 2013. https://www.who.int/publications/i/item/978-92-4-154837-3.

[7] Sands K , Carvalho MJ, Portal E et al Characterization of antimicrobial-resistant gram-negative bacteria that cause neonatal sepsis in seven low- and middle-income countries. Nat Microbiol 2021; 6: 512–23. 10.1038/s41564-021-00870-7 33782558 PMC8007471

[8] Investigators of the Delhi Neonatal Infection Study (DeNIS) collaboration . Characterisation and antimicrobial resistance of sepsis pathogens in neonates born in tertiary care centres in Delhi, India: a cohort study. Lancet Glob Heal 2016; 4: e752–60. 10.1016/S2214-109X(16)30148-6 27633433

[9] Labi AK , Obeng-Nkrumah N, Bjerrum S et al Neonatal bloodstream infections in a Ghanaian tertiary hospital: are the current antibiotic recommendations adequate? BMC Infect Dis 2016; 16: 598. 10.1186/s12879-016-1913-4 27776490 PMC5078915

[10] Bandyopadhyay T , Kumar A, Saili A et al Distribution, antimicrobial resistance and predictors of mortality in neonatal sepsis. J Neonatal Perinatal Med 2018; 11: 145–53. 10.3233/NPM-1765 29991144

[11] Jajoo M , Manchanda V, Chaurasia S et al Alarming rates of antimicrobial resistance and fungal sepsis in outborn neonates in north India. PLoS One 2018; 13: e0180705. 10.1371/journal.pone.0180705 29953451 PMC6023165

[12] Yadav NS , Sharma S, Chaudhary DK et al Bacteriological profile of neonatal sepsis and antibiotic susceptibility pattern of isolates admitted at Kanti Children’s Hospital, Kathmandu, Nepal. BMC Res Notes 2018; 11: 301. 10.1186/s13104-018-3394-6 29764503 PMC5952417

[13] Pokhrel B , Koirala T, Shah G et al Bacteriological profile and antibiotic susceptibility of neonatal sepsis in neonatal intensive care unit of a tertiary hospital in Nepal. BMC Pediatr 2018; 18: 208. 10.1186/s12887-018-1176-x 29950162 PMC6020420

[14] Chaurasia S , Sivanandan S, Agarwal R et al Neonatal sepsis in South Asia: huge burden and spiralling antimicrobial resistance. BMJ 2019; 364: K5314. 10.1136/bmj.k5314 30670451 PMC6340339

[15] Okomo U , Akpalu ENK, Le DK et al Aetiology of invasive bacterial infection and antimicrobial resistance in neonates in sub-Saharan Africa: a systematic review and meta-analysis in line with the STROBE-NI reporting guidelines. Lancet Infect Dis 2019; 19: 1219–34. 10.1016/S1473-3099(19)30414-1 31522858

[16] Folgori L , Ellis SJ, Bielicki JA et al Tackling antimicrobial resistance in neonatal sepsis. Lancet Glob Heal 2017; 5: e1066–8. 10.1016/S2214-109X(17)30362-5 29025624

[17] Kawaguchi H , Naito T, Nakagawa S et al BB-K 8, a new semisynthetic aminoglycoside antibiotic. J Antibiot (Tokyo) 1972; 25: 695–708. 10.7164/antibiotics.25.695 http://www.ncbi.nlm.nih.gov/pubmed/4568692. 4568692

[18] Ramirez MS , Tolmasky ME. Aminoglycoside modifying enzymes. Drug Resist Updat 2010; 13: 151–71. 10.1016/j.drup.2010.08.003 20833577 PMC2992599

[19] Darlow CA , da Costa RMA, Ellis S et al Potential antibiotics for the treatment of neonatal sepsis caused by multidrug-resistant bacteria. Pediatr Drugs 2021; 23: 465–84. 10.1007/s40272-021-00465-z PMC8418595 34435316

[20] Ito M , Ishigami T. The meaning of the development of flomoxef and clinical experience in Japan. Infection 1991; 19: 253–7. 10.1007/BF01645536 1783441

[21] Darlow CA , Docobo-Perez F, Farrington N et al Amikacin combined with fosfomycin for treatment of neonatal sepsis in the setting of highly prevalent antimicrobial resistance. Antimicrob Agents Chemother 2021; 65: e0029321. 10.1128/AAC.00293-21 33972238 PMC8373250

[22] Darlow CA , Farrington N, Johnson A et al Flomoxef and fosfomycin in combination for the treatment of neonatal sepsis in the setting of highly prevalent antimicrobial resistance. J Antimicrob Chemother 2022; 77: 1334–43. 10.1093/jac/dkac038 35170719 PMC9047679

[23] European Committee for Antimicrobial Susceptibility Testing (EUCAST) of the European Society of Clinical Microbiology and Infectious Diseases (ESCMID) . Determination of minimum inhibitory concentrations (MICs) of antibacterial agents by broth dilution. Clin Microbiol Infect 2003; 9: ix–xv. 10.1046/j.1469-0691.2003.00790.x 11168187

[24] EUCAST . Routine and extended internal quality control for MIC determination and disk diffusion as recommended by EUCAST. Version 11.0. 2021.

[25] Nagayama A , Yamaguchi K, Watanabe K et al Final report from the Committee on Antimicrobial Susceptibility Testing, Japanese Society of Chemotherapy, on the agar dilution method (2007). J Infect Chemother 2008; 14: 383–92. 10.1007/s10156-008-0634-Z 18936894

[26] Greco WR , Park HS, Rustum YM. Application of a new approach for the quantitation of drug synergism to the combination of cis-diamminedichloroplatinum and 1-θ-D-arabinofuranosylcytosine. Cancer Res 1990; 50: 5318–27. 2386940

[27] D’Argenio DZ , Schumitzky A, Wang X. ADAPT 5 user’s guide: pharmacokinetic/pharmacodynamic systems analysis software. Biomedical Simulations Resource, 2009.

[28] Viechtbauer W . Conducting meta-analyses in R with the metafor package. J Stat Softw 2010; 36: 1–48. 10.18637/jss.v036.i03

[29] Cadwell J . The hollow fiber infection model for antimicrobial pharmacodynamics and pharmacokinetics. Adv Pharmacoepidemiol Drug Saf 2012; S1:007. 10.4172/2167-1052.S1-007

[30] Ramos-Martín V , Johnson A, Livermore J et al Pharmacodynamics of vancomycin for CoNS infection: experimental basis for optimal use of vancomycin in neonates. J Antimicrob Chemother 2016; 71: 992–1002. 10.1093/jac/dkv451 26755499 PMC4790623

[31] Shionogi . Flumarin for intravenous injection SPC. 2009.

[32] EMC . Amikacin 250 mg/mL injection SPC. 2015. https://www.medicines.org.uk/emc/product/3784.

[33] Blaser J . In-vitro model for simultaneous simulation of the serum kinetics of two drugs with different half-lives. J Antimicrob Chemother 1985; 15: 125–30. 10.1093/jac/15.suppl_A.125 3980323

[34] Clarke JT , Libke RD, Regamey C et al Comparative pharmacokinetics of amikacin and kanamycin. Clin Pharmacol Ther 1974; 15: 610–6. 10.1002/cpt1974156610 4210297

[35] Sando M , Sato Y, Iwata S et al Protein binding ability of various antimicrobial drugs in neonates. Japanese J Chemother 2004; 52: 568–73.

[36] Sadouki Z , McHugh TD, Aarnoutse R et al Application of the hollow fibre infection model (HFIM) in antimicrobial development: a systematic review and recommendations of reporting. J Antimicrob Chemother 2021; 76: 2252–9. 10.1093/jac/dkab160 34179966 PMC8361333

[37] Anu K , Esther K, Dunn SJ et al Real-time sampling of travelers shows intestinal colonization by multidrug-resistant bacteria to be a dynamic process with multiple transient acquisitions. bioRxiv 2019: 827915. 10.1101/827915

[38] Neely MN , Van Guilder MG, Yamada WM et al Accurate detection of outliers and subpopulations with Pmetrics, a nonparametric and parametric pharmacometric modeling and simulation package for R. Ther Drug Monit 2012; 34: 467–76. 10.1097/FTD.0b013e31825c4ba6 22722776 PMC3394880

[39] Jett BD , Hatter KL, Huycke MM et al Simplified agar plate method for quantifying viable bacteria. Biotechniques 1997; 23: 648–50. 10.2144/97234bm22 9343684

[40] Hughes KM , Johnson PN, Anderson MP et al Comparison of amikacin pharmacokinetics in neonates following implementation of a new dosage protocol. J Pediatr Pharmacol Ther 2017; 22: 33–40. 10.5863/1551-6776-22.1.33 28337079 PMC5341529

[41] Bulitta JB , Hope WW, Eakin AE et al Generating robust and informative nonclinical *in vitro* and *in vivo* bacterial infection model efficacy data to support translation to humans. Antimicrob Agents Chemother 2019; 63: e02307-18. 10.1128/AAC.02307-18 30833428 PMC6496039

[42] EUCAST . Breakpoint tables for interpretation of MICs and zone diameters Version 10.0. 2020.

[43] Lee CH , Su LH, Tang YF et al Treatment of ESBL-producing *Klebsiella pneumoniae* bacteraemia with carbapenems or flomoxef: a retrospective study and laboratory analysis of the isolates. J Antimicrob Chemother 2006; 58: 1074–7. 10.1093/jac/dkl381 16971415

[44] Matsumura Y , Yamamoto M, Nagao M et al In vitro activities and detection performances of cefmetazole and flomoxef for extended-spectrum β-lactamase and plasmid-mediated AmpC β-lactamase-producing Enterobacteriaceae. Diagn Microbiol Infect Dis 2016; 84: 322–7. 10.1016/j.diagmicrobio.2015.12.001 26782634

[45] Lee CH , Chu C, Liu JW et al Collateral damage of flomoxef therapy: *in vivo* development of porin deficiency and acquisition of *bla*_DHA-1_ leading to ertapenem resistance in a clinical isolate of *Klebsiella pneumoniae* producing CTX-M-3 and SHV-5 β-lactamases. J Antimicrob Chemother 2007; 60: 410–3. 10.1093/jac/dkm215 17576696

[46] Jacoby GA , Carreras I. Activities of β-lactam antibiotics against *Escherichia coli* strains producing extended-spectrum β-lactamases. Antimicrob Agents Chemother 1990; 34: 858–62. 10.1128/AAC.34.5.858 2193623 PMC171706

[47] Jacoby GA . Ampc Β-lactamases. Clin Microbiol Rev 2009; 22: 161–82. 10.1128/CMR.00036-08 19136439 PMC2620637

[48] Thomson KM , Dyer C, Liu F et al Effects of antibiotic resistance, drug target attainment, bacterial pathogenicity and virulence, and antibiotic access and affordability on outcomes in neonatal sepsis: an international microbiology and drug evaluation prospective substudy (BARNARDS). Lancet Infect Dis 2021; 21: 1677–88. 10.1016/S1473-3099(21)00050-5 34384533 PMC8612937

[49] Vilken T , Xavier BB, Glupczynski Y et al In vitro activity of WHO recommended, widely used and potential novel antibiotics against *K. pneumoniae* and *E. coli* from neonatal sepsis in settings with high level of antimicrobial resistance: results of the global NeoOBS Study. *European Congress of Clinical Microbiology and Infectious Diseases, 23–26 April 2022, Lisbon, Portugal*. Abstract number 04940.

[50] Okada T , Furukawa S. Clinical evaluation of flomoxef in pediatrics and a study on the penetration into cerebrospinal fluid. Jpn J Antibiot 1987; 40: 1477–85. 10.7164/antibiotics.40.230 3430723

[51] Allegaert K , Scheers I, Adams E et al Cerebrospinal fluid compartmental pharmacokinetics of amikacin in neonates. Antimicrob Agents Chemother 2008; 52: 1934–9. 10.1128/AAC.01099-07 18378715 PMC2415815

[52] Pickering LK , Ericsson CD, Ruiz Palacios G, et al Intraventricular and parenteral gentamicin therapy for ventriculitis in children. Am J Dis Child 1978; 132: 480–3. 10.1001/archpedi.1978.02120300040007 645674

[53] Clumeck N , Thys JP, Vanhoof R et al Amoxicillin entry into human cerebrospinal fluid. Comparison with ampicillin. Antimicrob Agents Chemother 1978; 14: 531–2. 10.1128/AAC.14.4.531 102244 PMC352502

[54] Denis F , Cadoz M, Mounier M et al Spinal concentrations of amoxicillin in purulent meningitis in children. Pathol Biol (Paris) 1983; 31: 308–10. 6353322

[55] Kellogg JA , Ferrentino FL, Goodstein MH et al Frequency of low level bacteremia in infants from birth to two months of age. Pediatr Infect Dis J 1997; 16: 381–5. 10.1097/00006454-199704000-00009 9109140

[56] Dietzman DE , Fischer GW, Schoenknecht FD. Neonatal *Escherichia coli* septicemia-bacterial counts in blood. J Pediatr 1974; 85: 128–30. 10.1016/S0022-3476(74)80308-2 4604810

