# Supplementary material for: Assessment of flomoxef combined with amikacin in a hollow-fibre infection model for the treatment of neonatal sepsis in low- and middle-income healthcare settings
Source: J Antimicrob Chemother. 2022 Sep 30;77(12):3349–57. doi: 10.1093/jac/dkac323 (PMC9704437; doi:10.1093/jac/dkac323)
Supplement: dkac323_Supplementary_Data [file dkac323_supplementary_data.docx]

**Assessment of Flomoxef Combined with Amikacin in a Hollow Fiber Model for the Treatment of Neonatal Sepsis in Low- and Middle-Income Healthcare Settings – Supplementary Data**

**Supplementary text 1 – Bioanalysis method details**

**Flomoxef Bioanalysis.** The internal standard, Moxalactam (Cayman Chemicals, USA) was prepared in acetonitrile (25 mg/L, Fisher Scientific UK) and 200 μL was added to a 96-well protein precipitation plate (Phenomenex, Cheshire, UK). Fifty μL each of samples, blanks, calibrators in the range 0.1 – 50 mg/L and quality controls (0.75, 7.5 and 37.5 mg/L) were mixed with the internal standard on an orbital shaker. Liquid was drawn through the protein precipitation plate into a collection plate using a positive pressure manifold with water and water containing 0.1% formic acid (200 μL) added to each well, before sealing and mixing on an orbital shaker prior to analysis by liquid chromatography-tandem mass spectrometry (LC-MS/MS).

LC-MS/MS analysis was performed using an Agilent 1290 Infinity high-performance liquid chromatography (HPLC) system coupled to an Agilent 6420 triple-quadrupole mass spectrometer fitted with an electrospray source controlled using Agilent MassHunter data acquisition software (version B.06.00). Analytes were injected (4 μL) onto an Phenomenex Kinetex C18 column (100 A, 2.1 mm x 50 mm, 2.6 µm) and separated over a 4 min. gradient using a mixture of solvents A (LC-MS grade water containing 0.1% formic acid) and B (HPLC grade acetonitrile containing 0.1% formic acid). Separations were performed by applying a linear gradient of 95% to 5% solvent A over 2.50 mins at 0.6 mL/min followed by an equilibration step (1.5 mins at 95% solvent A).

The mass spectrometer was operated in positive ion mode using a Multiple Reaction Monitoring (MRM) method with the specified mass transitions and collision energies: flomoxef 497.0 > 199.0 (Ce 15 ev) and moxalactam 521.0 > 377.0 (Ce 10 ev). Mass spectrometry readouts were processed using Agilent MassHunter quantitative analysis (version B.05.02). Prior to sample analysis, the analytical method was validated to assess recovery and matrix effects, interday and intraday accuracy and precision, carryover, dilution integrity, stability in the matrix (4 h at room temperature and three-freeze thaw cycles), and processed sample stability (reinjection of extracts after 24 h).

The average recovery from matrix was 83%. The LLQ was defined as 0.1 mg/L and the LOD 0.05 mg/L. The inter- and intra-day %CV on the three QC levels ranged from 5.04% – 7.65% and -5.68% – 2.16% respectively. The analyte was found to be stable in all conditions described above.

**Amikacin Bioanalysis.** The internal standard, [^2^H_5_] amikacin (Alsachim, Illkirch-Graffenstaden, France) was prepared in acetonitrile plus 5% trichloroacetic acid (TCA) (25 mg/L, Fisher Scientific, UK) and 150 μL was added to a 96-well protein precipitation plate (Phenomenex, Cheshire, UK). Fifty μL each of samples, blanks, calibrators in the range 0.5 – 50 mg/L and quality controls (0.75, 7.5 and 37.5 mg/L) were mixed with the internal standard on an orbital shaker. Liquid was drawn through the protein precipitation plate into a collection plate using a positive pressure manifold. Samples were evaporated under nitrogen (40 L/min) followed by reconstitution in water (Fisher Scientific, UK) and 0.1% heptafluorobutyric acid [Sigma-Aldrich, UK] and mixed using an orbital shaker prior to analysis by LC-MS-MS.

LC-MS-MS analysis was performed using an Agilent 1290 Infinity HPLC coupled to an Agilent 6420 triple quadrupole mass spectrometer fitted with an electrospray source controlled using Agilent MassHunter Data Acquisition software (Ver B.06.00). Analytes were injected (5 μL) onto a Discovery® HS C18 HPLC Column (2.1 mm x 50 mm, 3 µm, 50°C) and separated over a 3.5 min. gradient using a mixture of solvents A (LC-MS grade water with 0.1% (v/v) heptafluorobutyric acid) and B (HPLC grade acetonitrile with 0.1% (v/v) heptafluorobutyric acid). Separations were performed by applying a linear gradient of 2% to 98% solvent B over 3 mins at 0.5 mL/min followed by an equilibration step (0.5 mins at 2% solvent B).

The mass spectrometer was operated in positive ion mode using a Multiple Reaction Monitoring (MRM) method with the specified mass transitions and collision energies: amikacin 586.4 > 163.2 (Ce 30 ev) and [^2^H_5_] amikacin 591.3 > 163.2 (Ce 30 ev). Mass spectrometry readouts were processed using Agilent Mass Hunter Quantitative Analysis (Ver B.05.02).

Prior to sample analysis, the analytical method was validated to assess recovery and matrix effects, inter- and intra-day accuracy and precision, carryover, dilution integrity, stability in matrix (4 hours at room temperature and 3 freeze thaw cycles) and processed sample stability (reinjection of extracts after 24hrs). The average recovery from matrix was 75.3%. The limit of quantification (LLQ) was defined as 0.5 mg/L and the limit of detection (LOD) 0.25 mg/L. The inter- and intra-day %CV on the three QC levels ranged from 2.5% – 5.7% and 2.9% – 6.41% respectively. The analyte was found to be stable in all conditions described above.

**Supplementary Text 2**

The structural model used in the population PK model of the 16-arm HFIM experiment took the form as follows:

$$[1]. \frac{dX1}{dt}=R\left( 1 \right)-\left( \frac{Cl1}{V1} \right)*X1$$

$$[2]. \frac{dX2}{dt}=R\left( 2 \right)-\left( \frac{Cl2}{V2} \right)*X2$$

$$[3a]. \frac{dX3}{dt}=X3*(Kgs*(1-\frac{\left( X3+X4+X5 \right)}{POPmax})-\frac{Kks*XMs}{(1+XMs)})$$

$$\left[ 3b \right]. if X1=0 then XMs=\left( \frac{\frac{X2}{V2}}{E{50}_{2}s} \right)^{H2s}$$

$$\left[ 3c \right]. if X2=0 then XMs=\left( \frac{\frac{X1}{V1}}{{E50}_{1}s} \right)^{H1s}$$

$$\left[ 3d \right]. Else: \frac{(\frac{X1}{V1})}{{E50}_{1}s*{XMs}^{H1s}}+\frac{(\frac{X2}{V2})}{{E{50}_{2}s*XMs}^{H2s}}+\frac{{(\alpha}_{s}* \frac{X1}{V1}*\frac{X2}{V2})}{{E50}_{1}s*E{50}_{2}s*{XMs}^{\frac{H1s+H2s}{2}}}=1$$

$$\left[ 4a \right]. \frac{dX4}{dt}=X4*(Kgr1*(1-\frac{\left( X3+X4+X5 \right)}{POPmax})-\frac{Kkr1*XMr1}{(1+XMr1)})$$

$$\left[ 4b \right]. if X1=0 then XMr1=\left( \frac{\frac{X2}{V2}}{E{50}_{2}r1} \right)^{H2r1}$$

$$\left[ 4c \right]. if X2=0 then XMr1=\left( \frac{\frac{X1}{V1}}{{E50}_{1}r1} \right)^{H1r1}$$

$$\left[ 4d \right]. Else: \frac{(\frac{X1}{V1})}{{E50}_{1}r1*{XMr1}^{H1r1}}+\frac{(\frac{X2}{V2})}{{E{50}_{2}r1*XMr1}^{H2r1}}+\frac{{(\alpha}_{r1}* \frac{X1}{V1}*\frac{X2}{V2})}{{E50}_{1}r1*E{50}_{2}r1*{XMr1}^{\frac{H1r1+H2r1}{2}}}=1$$

$$[5a]. \frac{dX5}{dt}=X5*(Kgr2*(1-\frac{\left( X3+X4+X5 \right)}{POPmax})-\frac{Kkr2*XMr2}{(1+XMr2)})$$

$$\left[ 5b \right]. if X1=0 then XMr2=\left( \frac{\frac{X2}{V2}}{E{50}_{2}r2} \right)^{H2r2}$$

$$\left[ 5c \right]. if X2=0 then XMr2=\left( \frac{\frac{X1}{V1}}{{E50}_{1}r2} \right)^{H1r2}$$

$$\left[ 5d \right]. Else: \frac{(\frac{X1}{V1})}{{E50}_{1}r2*{XMr2}^{H1r2}}+\frac{(\frac{X2}{V2})}{{E{50}_{2}r2*XMr2}^{H2r2}}+\frac{{(\alpha}_{r2}* \frac{X1}{V1}*\frac{X2}{V2})}{{E50}_{1}r2*E{50}_{2}r2*{XMr2}^{\frac{H1r2+H2r2}{2}}}=1$$

Equations 1 and 2 model the mass of flomoxef (X1) and amikacin (X2) over time (t). R(1) and R(2) refer the rate of addition of each drug to the HFIM system; Cl1 and Cl2 refer to the clearance of each drug; and V1 and V2 refer to the volume of distribution.

Equations 3, 4 and 5 model the bacterial populations that are fully susceptible (X3), resistant to flomoxef (X4), and resistant to amikacin (X5) (as quantified by bacterial growth on 4xMIC agar plates) over time (t). All equations are identical except for the suffixes on equation terms associated with each population (s, r1 and r2, respectively).

Equation 3a-5a model the growth bacterial population and kill due any present antibiotics. Kgs, Kgr1 and Kgr2 are the growth constant for each population; POPmax is the maximum bacterial population concentration within the HFIM; and Kks, Kkr1 and Kkr2 are the kill constants for each population.

XMs, XMr1 and XMr2 are conditional composite terms. If X1 or X2 = 0 (i.e., one drug is absent), then equations 3-5b-c define these functions, where H1 and H2 refer to the Hill constant for flomoxef and amikacin suffixed by the relevant population marker (s, r1 or r2) and E50_1_ and E50_2_ refer to the concentrations of flomoxef and amikacin required to achieve 50% of maximal efficacy in each bacterial population. In these circumstances, equations 3-5a describe a standard Emax model.

When X1 > 0 and X2 > 0 (i.e., either drug is present), equations 3-5d define XMs, XMr1 and XMr2. These equations replicate Greco’s model of synergy ^52^, with α_s_, α_r1_ and α_r2_ representing the interaction term for flomoxef and amikacin in each bacterial population. Under these conditions, XMs, XMr1 and XMr2 represent the composite term (E/(Econ-E)), where E is the measured effect of the present drugs and Econ is the control response. As the equations 3-5d are in an unclosed form, the values of XMs, XMr1 and XMr2 were determined via a Nelder-Mead algorithm.

**Supplementary Tables**

| Species | Isolate | Known Resistance Mechanisms | Source | Flomoxef MIC (mg/L) | Amikacin MIC  (mg/L) |
| --- | --- | --- | --- | --- | --- |
| *E. coli* | NCTC 13352* | TEM-10 | PHE | 0.125 | 2 |
| *E. coli* | NCTC 13353* | CTX-M-15 | PHE | 0.125 | 16 |
| *E. coli* | NCTC 13451* | CTX-M-15, OXA-1, TEM-1, aac6'-lb-cr, mph(A), catB4, tet(A), dfrA7, aadA5, sulI | PHE | 0.125 | 16 |
| *E. coli* | SPT 719* | SHV ESBL, TEM ESBL | JMI | 0.25 | 8 |
| *E. coli* | SPT 717 | CMYII | JMI | >32 | 4 |
| *E. coli* | SPT 731^†^ | CTX-M-1, TEM, ST131, O25b | JMI | 0.125 | 16 |
| *E. coli* | I1025*^†^ | ESBL, Fos | University of Birmingham | 8 | 4 |
| *E. coli* | I1057* | CTX-M-15, CMY-23, FQ-resistant | University of Birmingham | 0.0625 | 32 |
| *E. coli* | I779* | CTX-M-14 | University of Birmingham | 0.25 | 4 |
| *E. coli* | ATCC BAA2523* | OXA-48 | LGC Standards | 0.5 | 8 |
| *E. coli* | ST195^†‡^ | CTX-M-14 | University of Birmingham | 0.125 | 4 |
| *K. pneumoniae* | ATCC 700603 | SHV-18 ESBL | LGC Standards | 0.25 | 2 |
| *K. pneumoniae* | NCTC 13438 | KPC3 | PHE | >32 | >32 |
| *K. pneumoniae* | 1091463 | SHV-OSBL, TEM-OSBL, CTX-M-3 | IHMA | 8 | >32 |
| *K. pneumoniae* | H207 | Unspecified ESBL | University of Birmingham | 0.125 | 2 |
| *K. pneumoniae* | 1256506^†^ | SHV-OSBL; TEM-OSBL; CTX-M-2; CMY-2 | IHMA | 32 | 2 |
| *K. pneumoniae* | 1216477 | SHV-OSBL, TEM-OSBL, CTX-M-15 | IHMA | 0.25 | 8 |
| *K. pneumoniae* | 1237221 | SHV-OSBL, CTX-M-15 | IHMA | 0.25 | 8 |
| *K. pneumoniae* | 1280740*^†^ | SHV-OSBL, TEM-OSBL, CTX-M-15, DHA-1 | IHMA | 32 | 4 |
| *K. pneumoniae* | 1203217^†^ | SHV-12, CTX-M-9, OXA-48 | IHMA | 0.5 | 1 |
| *K. pneumoniae* | SPT 723* | CP_CTX-M_Group1, CP_SHV_ESBL, CP_SHV_WT, CTX-M-15 ,OXA-1/30, SHV-5 | JMI Laboratories | 0.25 | 4 |
| MRSA | NCTC 13656* | MecA | PHE | 4 | 4 |
| MRSA | NCTC 13435* | MecA | PHE | 1 | 2 |
| MRSA | NCTC 13616 | MecA | PHE | 4 | 2 |
| MRSA | NCTC 13626 | MecA | PHE | 16 | >32 |
| MRSA | NCTC 13813* | MecA | PHE | 2 | >32 |
| MRSA | F656 (USA300)* | MecA | University of Birmingham | 4 | 2 |
| MRSA | F40* | MecA | University of Birmingham | 0.25 | 4 |
| MRSA | F89 | MecA | University of Birmingham | 4 | 2 |
| MRSA | F82 | MecA | University of Birmingham | 0.5 | >32 |
| MRSA | F471 | MecA | University of Birmingham | 4 | 4 |
| *S. agalactiae* | NCTC 14091 | None | PHE | 0.5 | >32 |
| *S. agalactiae* | NCTC 14093 | None | PHE | 0.25 | >32 |
| *S. agalactiae* | NCTC 14095 | None | PHE | 0.25 | >32 |
| *S. agalactiae* | NCTC 14092 | None | PHE | 0.25 | >32 |
| *S. agalactiae* | 313566 (C13) | None | Royal Liverpool Hospital | 0.25 | >32 |
| *S. agalactiae* | 412909 (C14) | None | Royal Liverpool Hospital | 0.25 | >32 |
| *S. agalactiae* | 529312 (C15) | None | Royal Liverpool Hospital | 0.125 | >32 |
| *S. agalactiae* | 629129 (C16) | None | Royal Liverpool Hospital | 0.25 | >32 |
| *S. agalactiae* | 729701 (C17) | None | Royal Liverpool Hospital | 0.0625 | 16 |
| *S. agalactiae* | 824843 (C18)* | None | Royal Liverpool Hospital | 0.25 | 32 |

Table S1 : Details of isolates used in initial MIC assays. * denotes strains selected for assessment in checkerboard assays. ^†^ denotes strain used in HFIM experiments. ^‡^ denotes strains used only in HFIM; not included in the panel of strains for MIC determination. IHMA = International Health Management Associates; PHE = Public Health England.

**Supplementary Figures**

*
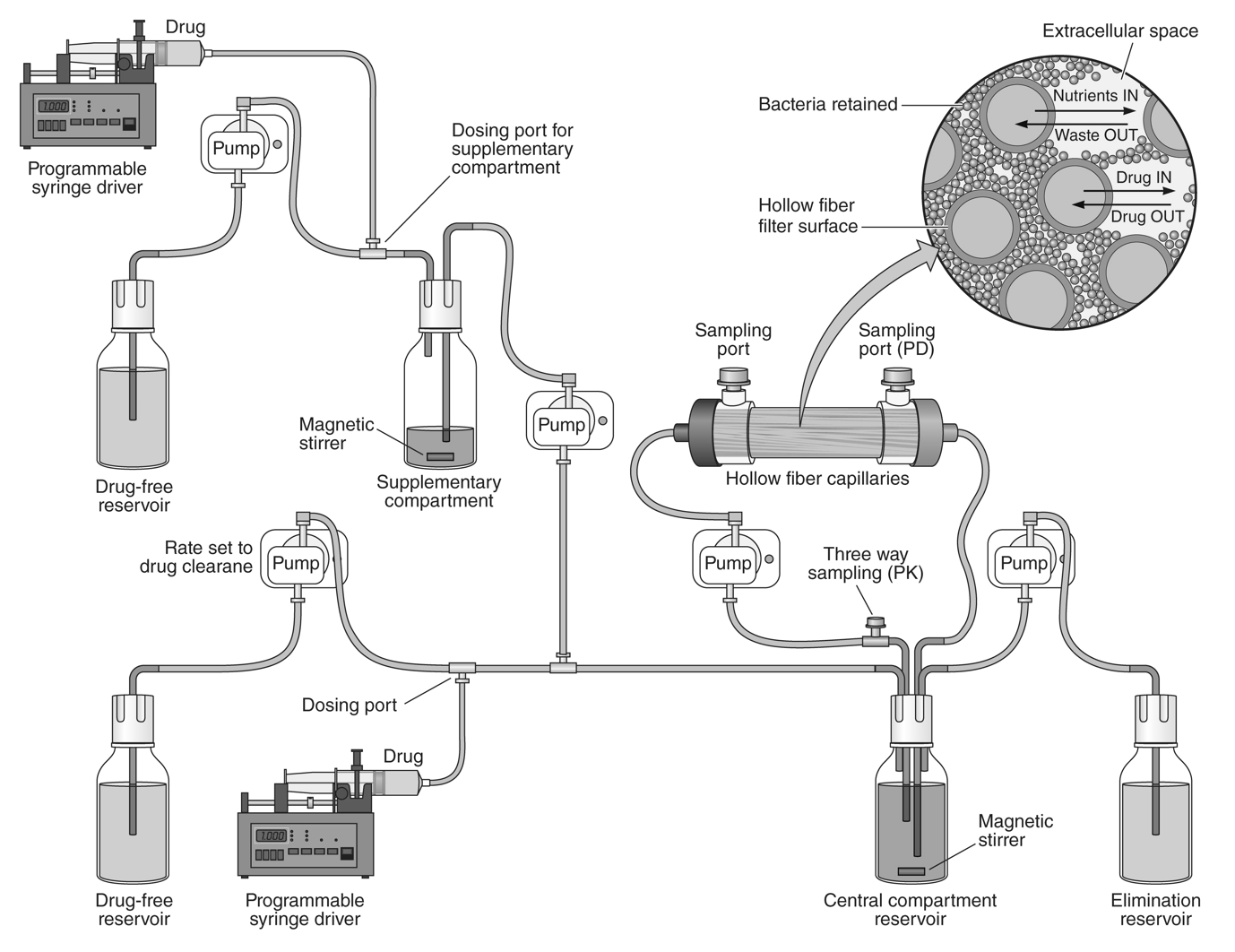
*

*Figure S1 – Schematic configuration of the HFIM with two agents. The supplementary compartments are omitted in arms with monotherapy.*


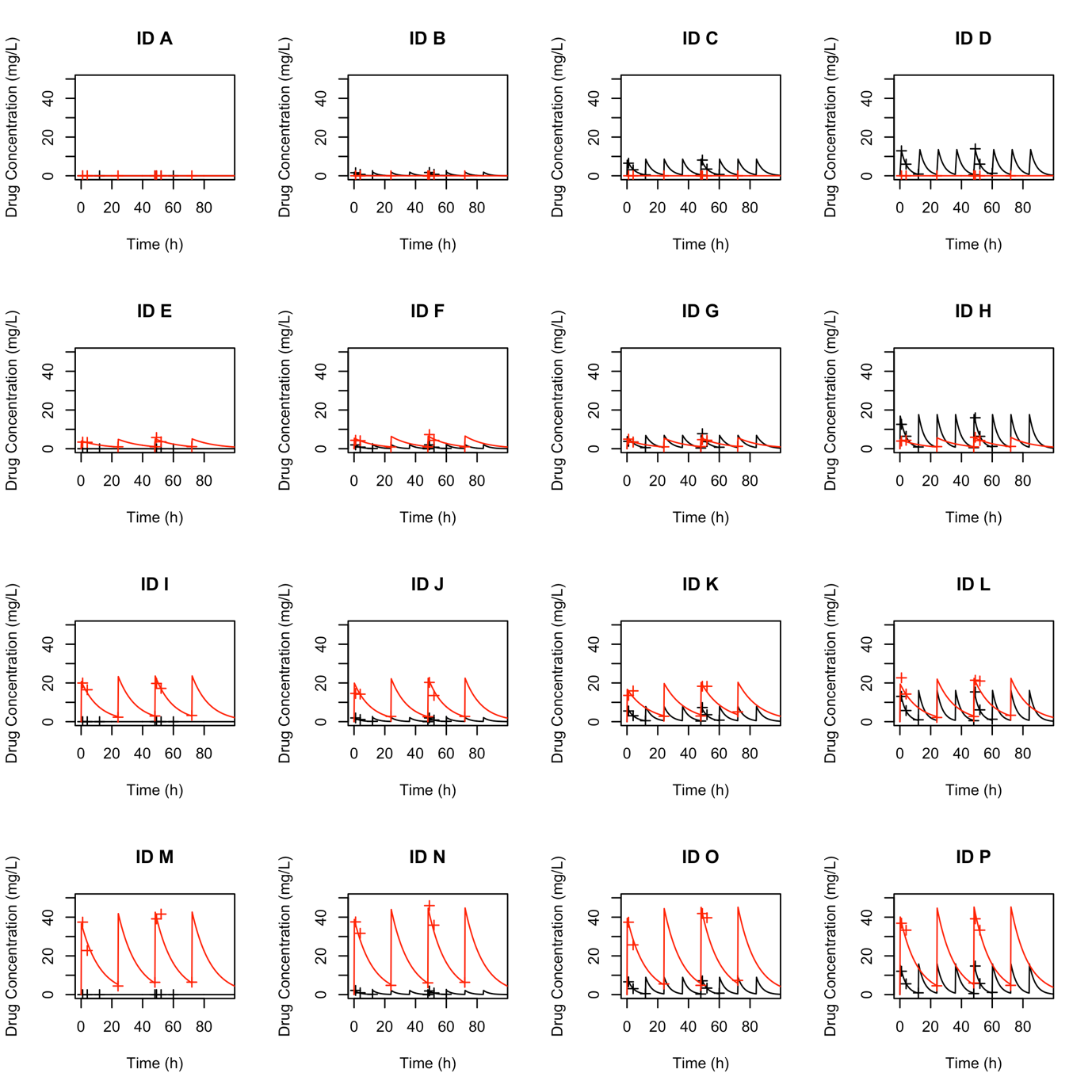


*Figure S2 – Modelled time-concentration profiles (solid lines) with overlaid drug measurements (+) for amikacin (red) and flomoxef (black) in the 16-arm HFIM experiment. ID A – P correspond to arms 1-16 respectively.*

*
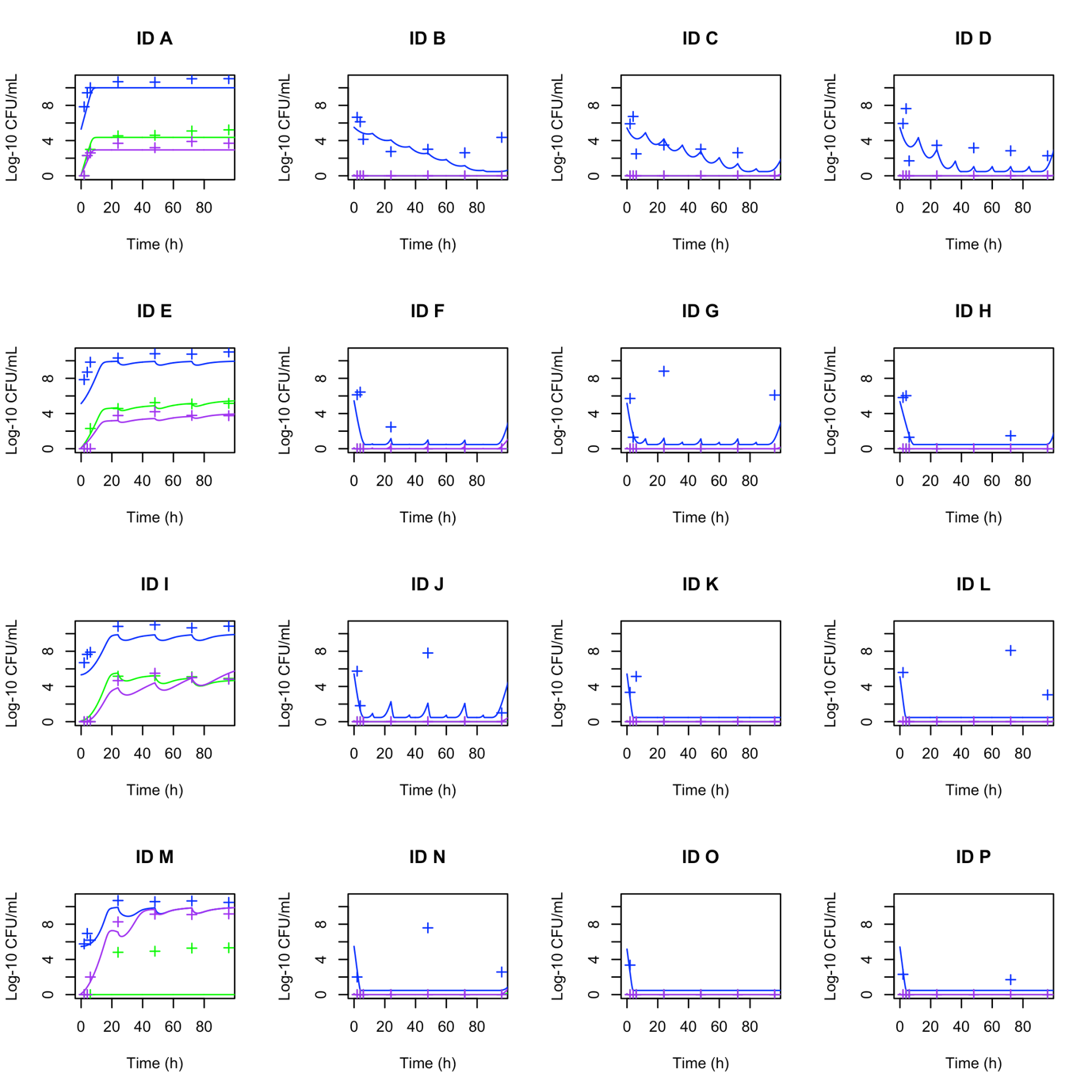
*

*Figure S3 – Modelled pharmacodynamic profiles (solid lines) with overlaid bacterial quantification (+) for total bacterial counts (blue), amikacin-resistant bacterial counts (magenta) and flomoxef-resistant bacterial counts (green). ID A – P correspond to arms 1-16 respectively. N.B. as noted in Table 3, the modelling of the flomoxef-resistant population is poor due to the lack of flomoxef-resistant populations emerging in any flomoxef-containing arm, and we therefore cannot comment on the effect of the combination on prevention of emergence of resistance to flomoxef.*

**
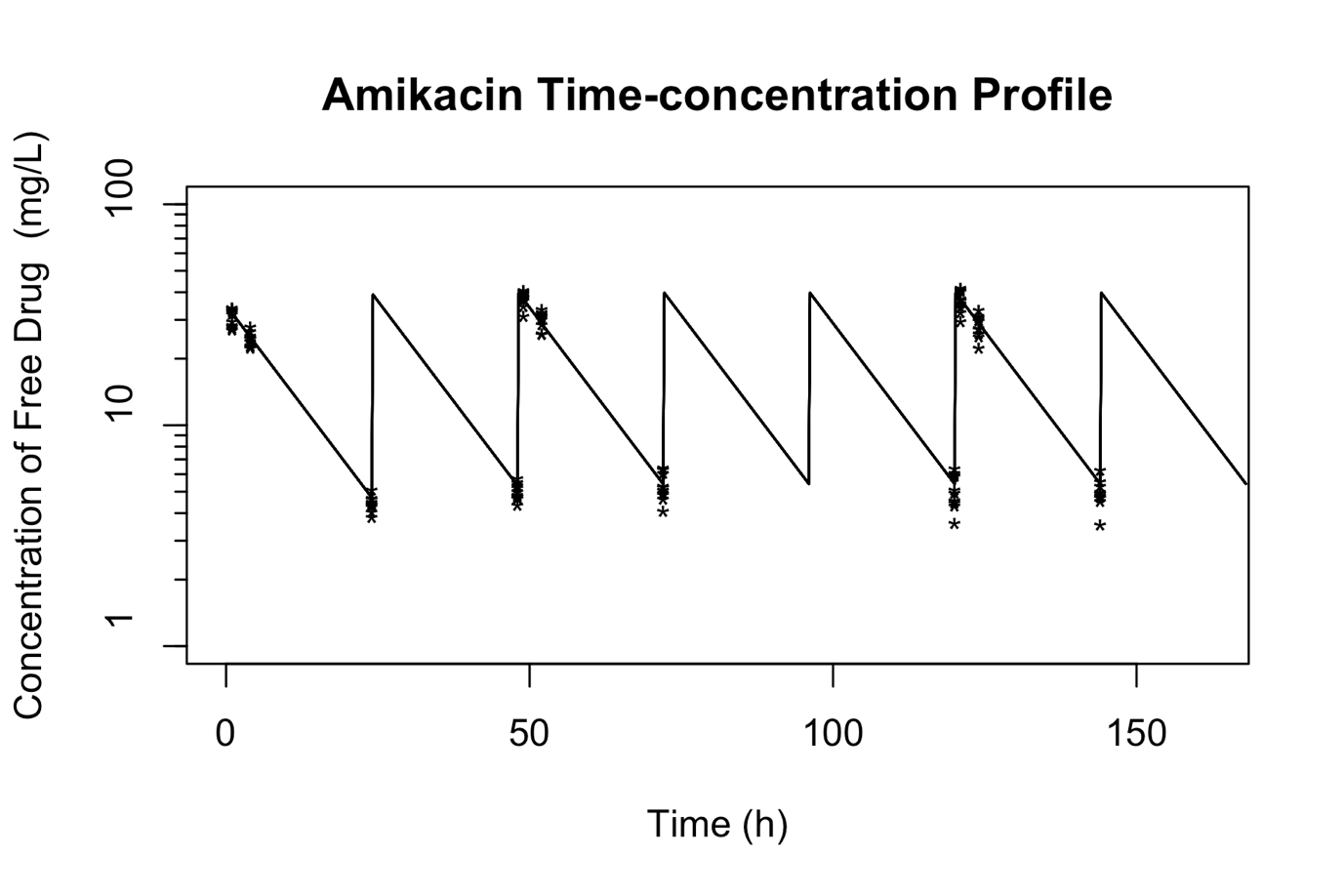
**

**
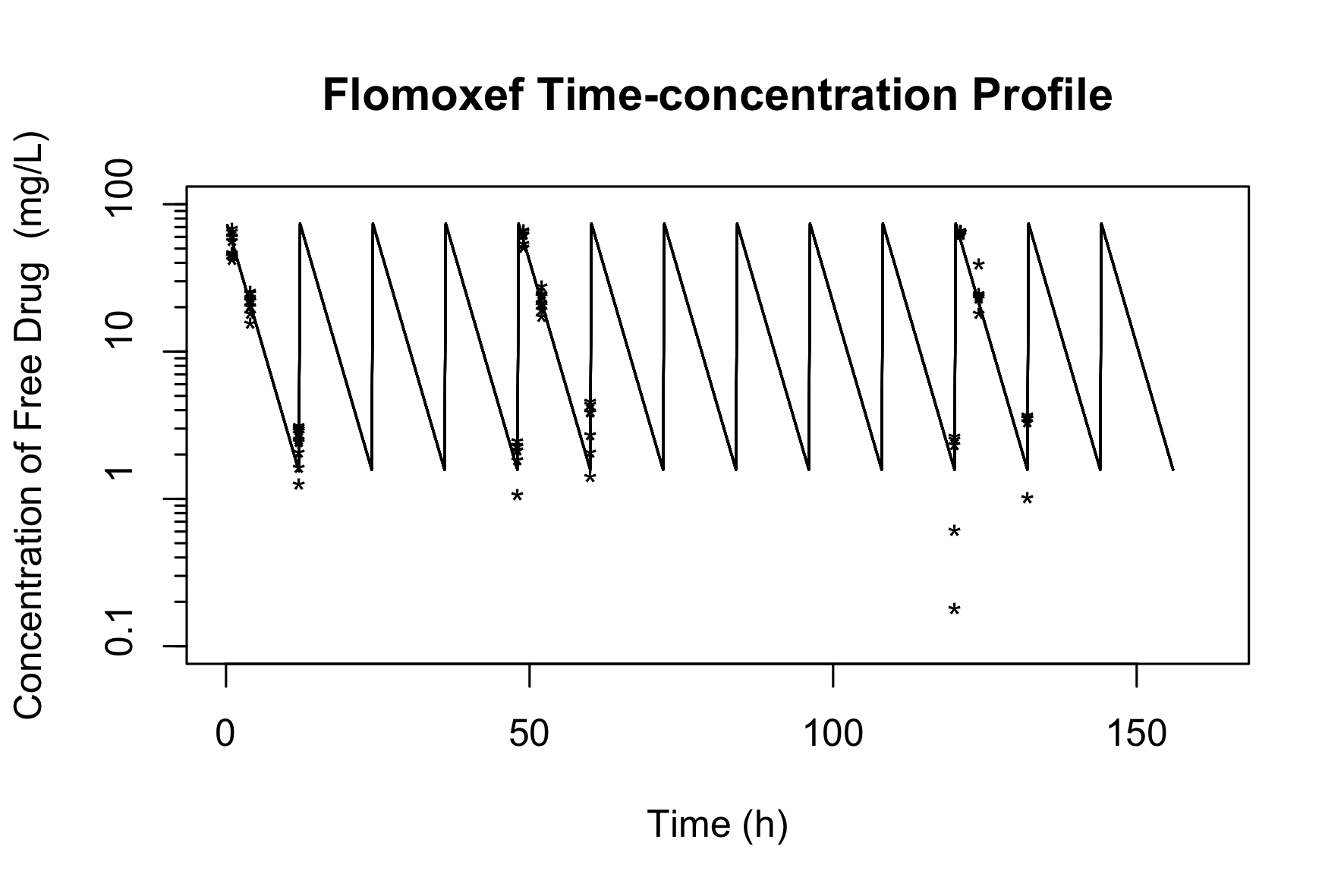
**

*Figure S4 – Modelled time concentrations (solid line) with overlaid measurement drug concentrations (*) for the HFIM experiments replicating clinically relevant drugs exposures for amikacin (top) and flomoxef (bottom). N.B. flomoxef drug concentration measurements below the limit of detection are omitted from the overlay, but still incorporated in the model.*


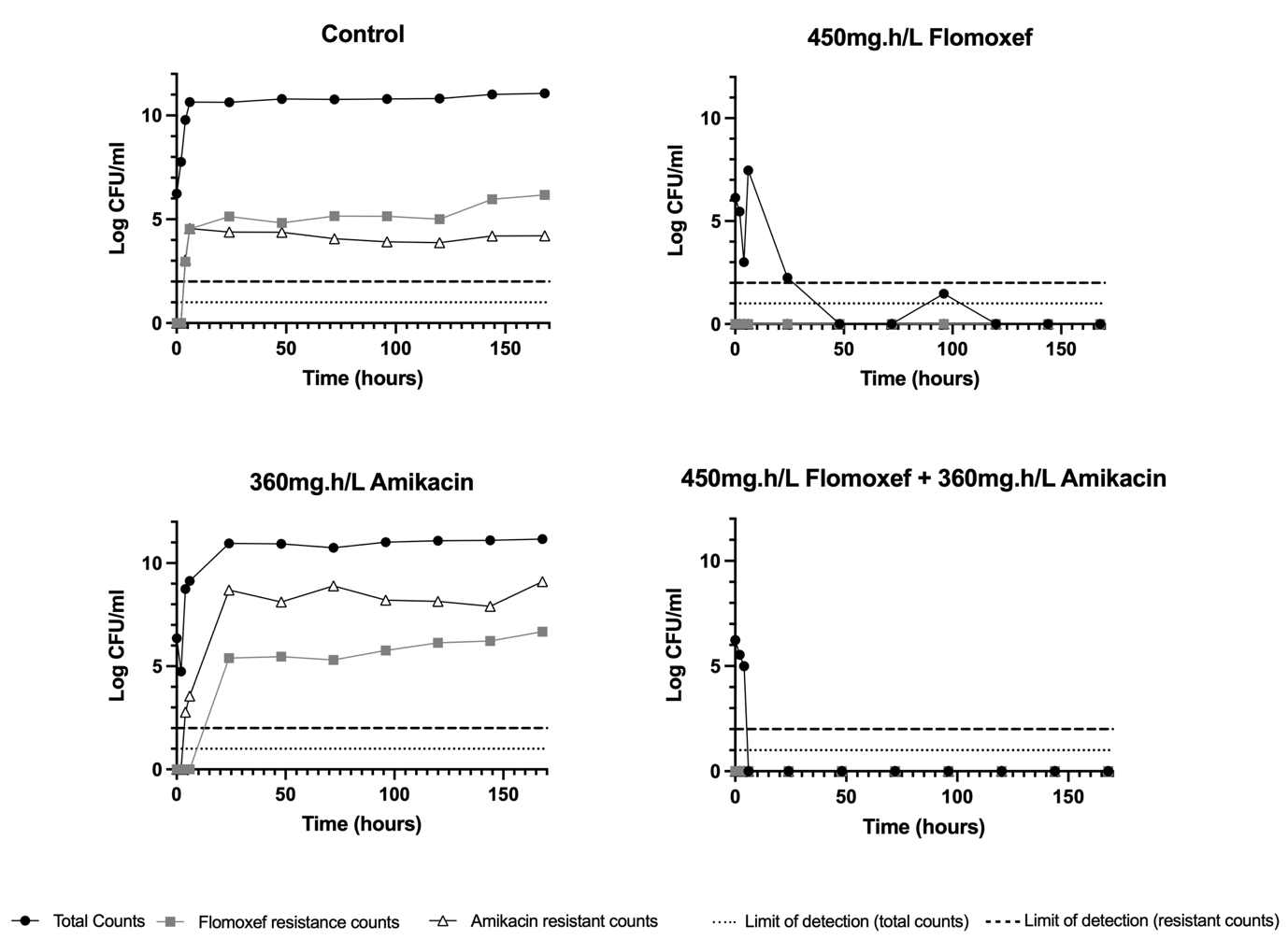


Figure S5 – HFIM pharmacodynamic results from 2x2 dosing matrix for E. coli SPT731 strain (Flomoxef MIC 0.125mg/L, Amikacin MIC 16mg/L)


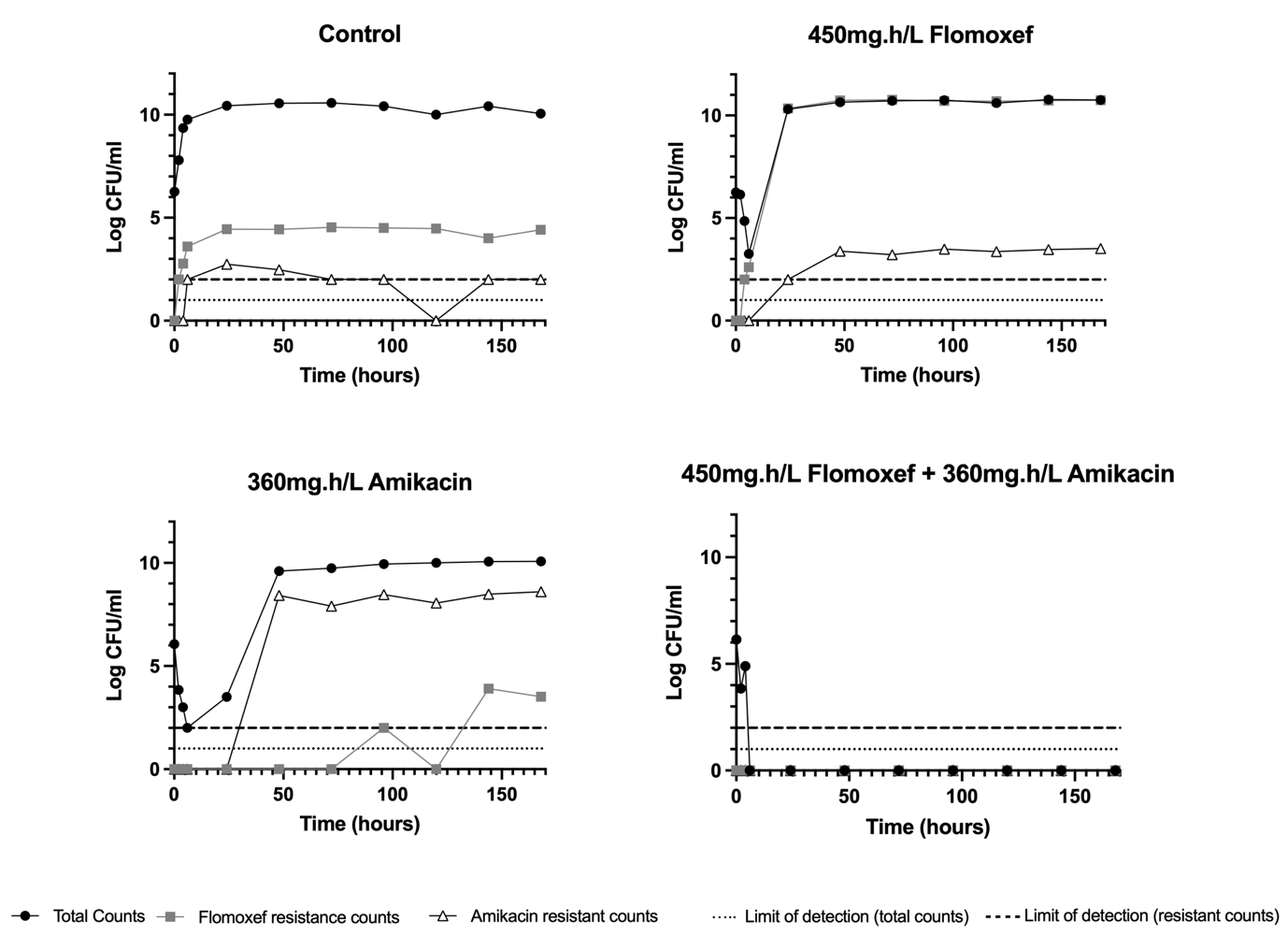


Figure S6 – HFIM pharmacodynamic results from 2x2 dosing matrix for K. pneumoniae 1203217 strain (Flomoxef MIC 0.5mg/L, Amikacin MIC 1mg/L)


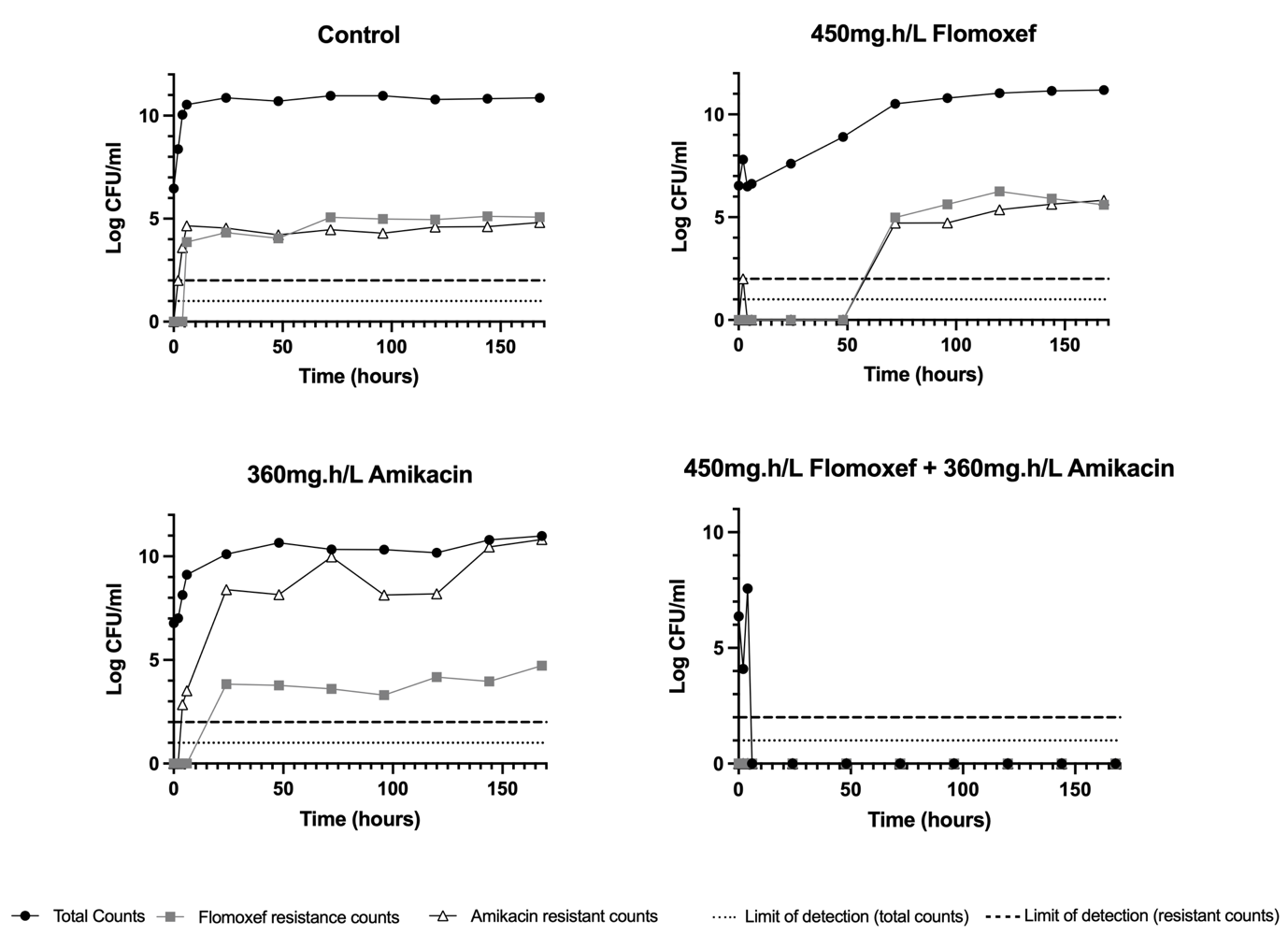


Figure S7 – HFIM pharmacodynamic results from 2x2 dosing matrix for E. coli I1025 strain (Flomoxef MIC 8mg/L, Amikacin MIC 4mg/L)


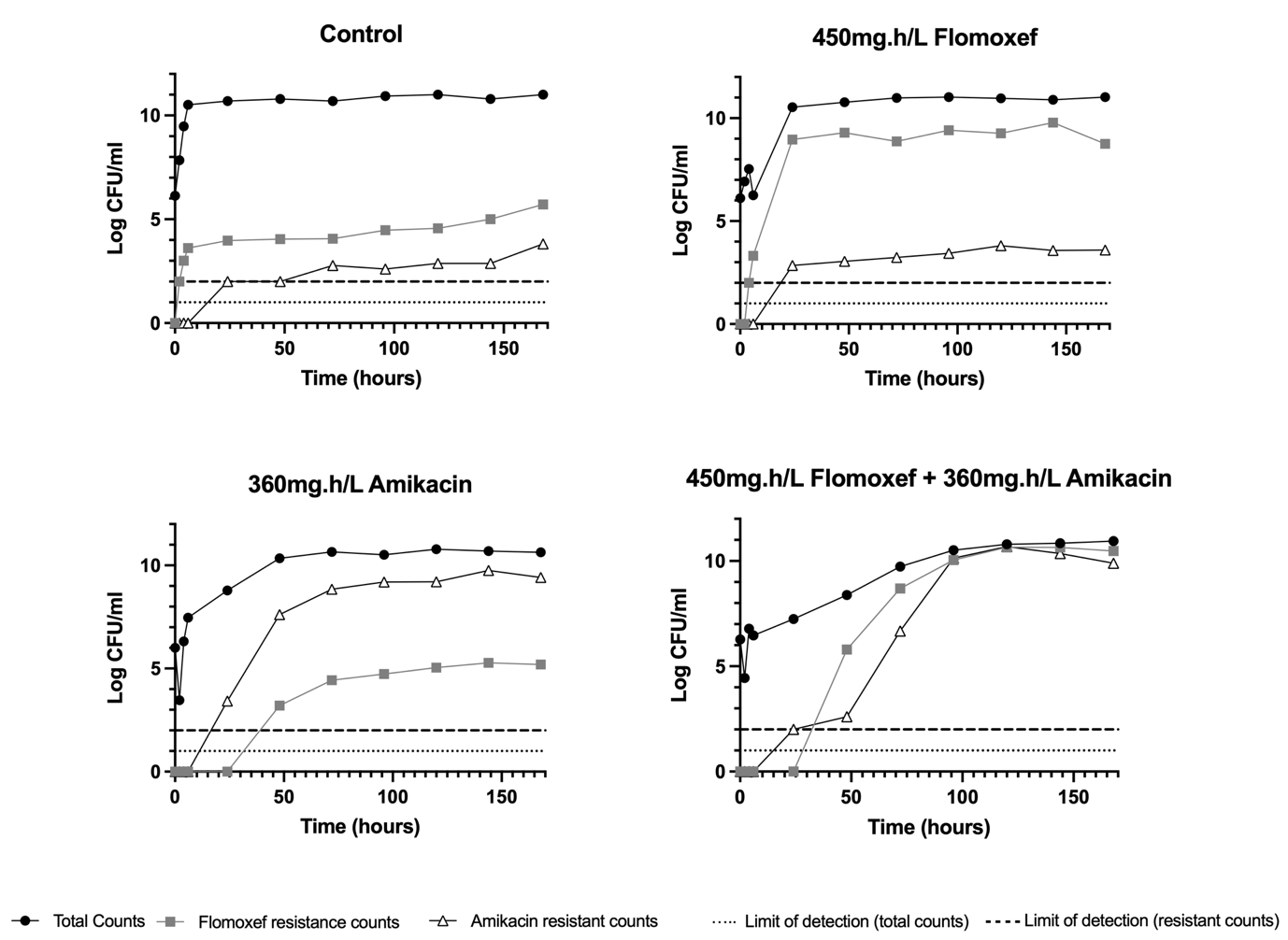


Figure S8 – HFIM pharmacodynamic results from 2x2 dosing matrix for K. pnueumoniae 1256506 strain (Flomoxef MIC 32mg/L, Amikacin MIC 2mg/L)


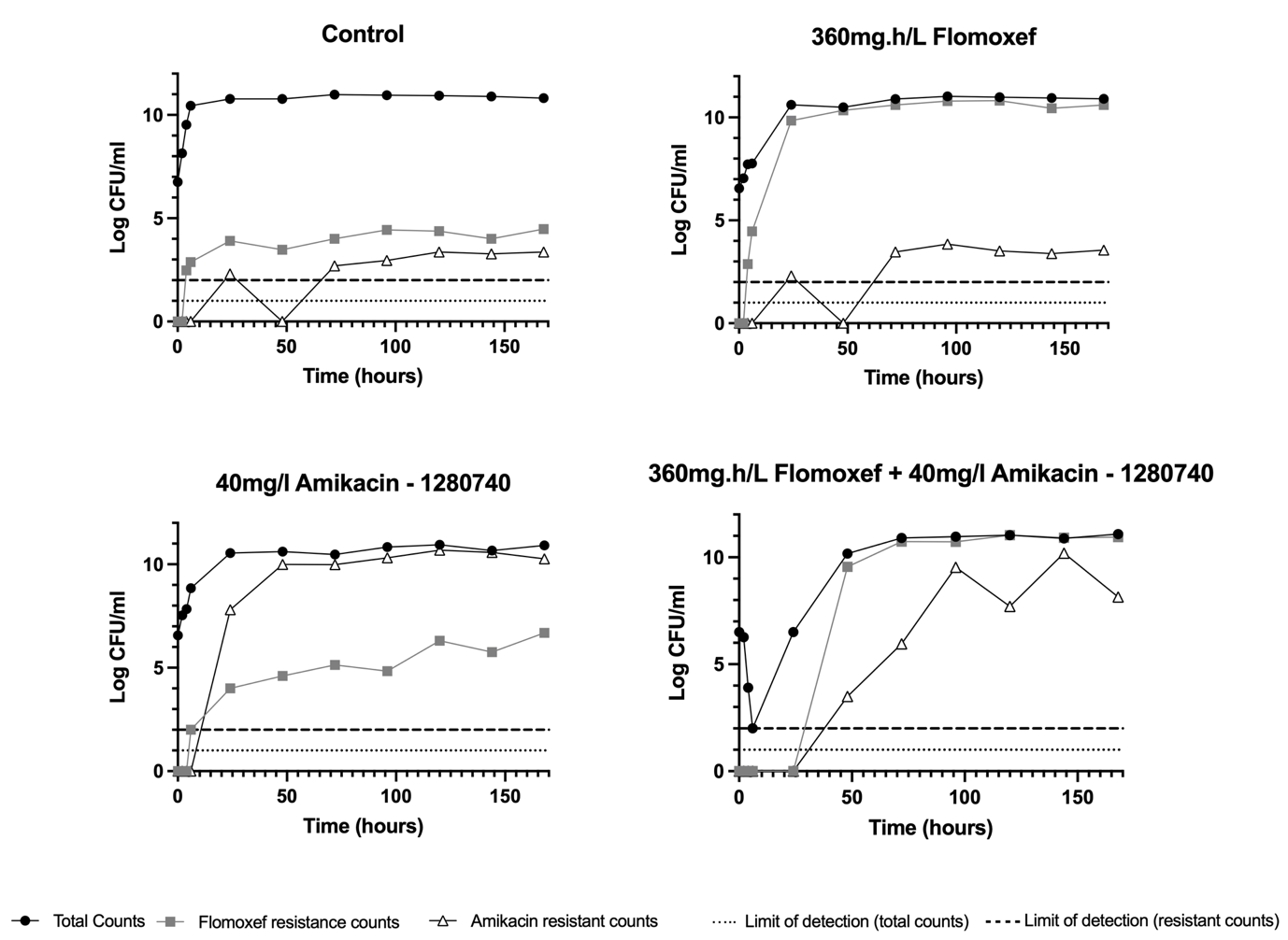


Figure S9 – HFIM pharmacodynamic results from 2x2 dosing matrix for K. pnueumoniae 1280740 strain (Flomoxef MIC 32mg/L, Amikacin MIC 4mg/L)
